# Utility of Metagenomic Next-Generation Sequencing for Characterization of HIV and Human Pegivirus Diversity

**DOI:** 10.1371/journal.pone.0141723

**Published:** 2015-11-23

**Authors:** Ka-Cheung Luk, Michael G. Berg, Samia N. Naccache, Beniwende Kabre, Scot Federman, Dora Mbanya, Lazare Kaptué, Charles Y. Chiu, Catherine A. Brennan, John Hackett

**Affiliations:** 1 Abbott Diagnostics, Infectious Disease Research, Abbott Park, Illinois, United States of America; 2 Department of Laboratory Medicine, University of California San Francisco, San Francisco, California, United States of America; 3 UCSF-Abbott Viral Diagnostics and Discovery Center, San Francisco, California, United States of America; 4 Université de Yaoundé 1, Yaoundé, Cameroon; 5 Université des Montagnes, Bangangté, Cameroon; 6 Department of Medicine, Division of Infectious Diseases, University of California San Francisco, San Francisco, California, United States of America; St. James School of Medicine, ANGUILLA

## Abstract

Given the dynamic changes in HIV-1 complexity and diversity, next-generation sequencing (NGS) has the potential to revolutionize strategies for effective HIV global surveillance. In this study, we explore the utility of metagenomic NGS to characterize divergent strains of HIV-1 and to simultaneously screen for other co-infecting viruses. Thirty-five HIV-1-infected Cameroonian blood donor specimens with viral loads of >4.4 log_10_ copies/ml were selected to include a diverse representation of group M strains. Random-primed NGS libraries, prepared from plasma specimens, resulted in greater than 90% genome coverage for 88% of specimens. Correct subtype designations based on NGS were concordant with sub-region PCR data in 31 of 35 (89%) cases. Complete genomes were assembled for 25 strains, including circulating recombinant forms with relatively limited data available (7 CRF11_cpx, 2 CRF13_cpx, 1 CRF18_cpx, and 1 CRF37_cpx), as well as 9 unique recombinant forms. HPgV (formerly designated GBV-C) co-infection was detected in 9 of 35 (25%) specimens, of which eight specimens yielded complete genomes. The recovered HPgV genomes formed a diverse cluster with genotype 1 sequences previously reported from Ghana, Uganda, and Japan. The extensive genome coverage obtained by NGS improved accuracy and confidence in phylogenetic classification of the HIV-1 strains present in the study population relative to conventional sub-region PCR. In addition, these data demonstrate the potential for metagenomic analysis to be used for routine characterization of HIV-1 and identification of other viral co-infections.

## Introduction

Molecular characterization of human immunodeficiency virus type 1 (HIV-1) has revealed an exceptional level of sequence diversity [1]. Several factors contribute to the overall genetic complexity, including high replication rates in an infected individual, error-prone replication by viral reverse transcriptase, and frequent inter-subtype recombination in high prevalence populations where more than two HIV strains are co-circulating. Phylogenetic analysis of full-length genomic sequences has classified HIV-1 into four distinct and highly divergent groups: M (major), O (outlier), N (non-M, non-O), and P, with group M strains, representing the pandemic branch, subdivided into nine subtypes (A-D, F-H, J and K). In addition to pure subtypes, more than 70 circulating recombinant forms (CRFs) of HIV-1 have also been described [2].

While a limited number of subtypes and CRFs predominate in any given geographical region, global diversification of HIV is continually being driven by the movement of people around the world and societal changes. Over the past 20 years, Europe and the United States, where HIV-1 subtype B infections are predominant, have seen a gradual increase in non-subtype B infections, primarily due to immigration. In France, non-B strains now account for approximately 50% of newly diagnosed HIV infections [3]. In the U.S., non-B infections have slowly increased from less than 1% before 1996 to approximately 4% by 2011 [4–6]. Social upheaval following the collapse of the Soviet Union also caused a rapid increase in HIV infections in the former Soviet Union (FSU) countries. In Russia, the number of infections increased from approximately 1000 in 1995 to greater than 255,000 by 2003 [7]. This HIV outbreak, driven by injection drug use, resulted in the emergence of CRF03_AB, a recombinant between the predominant subtype A strain in the FSU countries and subtype B [8]. CRF03_AB spread rapidly and by 2003 accounted for 4% of HIV infections in Russia, comprising 97% of the HIV infections in the Kaliningrad province [7]. These examples illustrate the dynamic nature of the HIV epidemic and the need for continuing viral surveillance.

The inherent capacity to generate sequence variation confers HIV with an ability to adapt to selective pressures applied by the host immune system and has immediate ramifications for pathogenesis including transmission, response to antiretroviral therapy, drug resistance, escape mutants and disease progression [1, 9]. The rapid evolution of HIV-1 also has significant implications from the perspective of screening, diagnostic testing, patient monitoring (e.g. viral load assays), and vaccine development. Surveillance is essential to monitor global diversification of HIV and to identify newly emerging strains. Typically surveillance has been conducted based on subtype-specific peptide immunoassays [10], heteroduplex mobility assays [11] or by Sanger sequencing of viral sub-genomic region(s) from many specimens [12] or the complete genome for a select few [13]. Next-generation sequencing (NGS), with unprecedented depth and coverage at a fraction of the cost and time of traditional sequencing, allows several specimens to be sequenced in parallel and thus has the potential to be leveraged to conduct viral surveillance [14]. Viral NGS can be applied for surveillance using target-specific [15] or unbiased metagenomic approaches [16]. By generating cDNA libraries using random priming, one can recover the sequence of entire genomes directly from primary clinical specimens. Thus, metagenomic NGS can accurately identify any particular HIV strain in a specimen, as well as the presence of additional co-infecting pathogens such as human pegivirus (HPgV, formerly GBV-C) [17], a common non-pathogenic virus that has been controversially linked to delayed progression to AIDS in the setting of co-infection with HIV-1 [18–20].

Considering the origins of the HIV epidemic, the population’s proximity to non-human primates, and the high level of strain diversity, Cameroon represents a logical site to conduct HIV surveillance [21, 22]. In Cameroon, the prevalence of HIV infection is estimated to be 4–5% in the adult population [23–25]. The Cameroonian HIV epidemic is notable for its high level of strain diversity [12, 13]. HIV-1 group M accounts for approximately 98% of infections, with CRF02 _AG being the majority (58%) strain present in the population [12]. All HIV-1 subtypes, a wide range of complex circulating recombinant forms (CRFs) and unique recombinant forms (URFs) of HIV-1 circulate in the population, with secondary recombination adding to the sizeable level of strain diversity [26]. Cameroon holds the distinction of being endemic for group O (accounts for 1–2% of infections; [12]), the rare group N [27, 28], the recently described group P [29, 30], and non-human primates that harbor the simian immunodeficiency viruses (SIV) most closely related to the HIV-1 groups [31–34]. In the current study, we apply NGS for full-genome viral sequencing and characterization of HIV-1 recombinant strains and HPgV from blood donors in Cameroon.

## Materials and Methods

### Selected specimens

This study was approved by the National Ethics Committee of Cameroon (Prof. Same Ekobo, Silvie Kwedi Nolna, Dr. Marceline Diuidje Ngounoue, Prof. Charles Fokunang, Dr. Chi Primus Che, Timoleon Tchuikam, Dr. Jerome Ateudjieu, Mireille Ndje Ndje, Gisele Magne). Written informed consent was obtained for all subjects. Specimens for this study were selected from HIV-1-infected blood donations collected in Douala and Yaoundé, Cameroon between 2002 and 2011. Available demographic data on donors is in S1 Table. The HIV-1 strain present in each blood donation was initially determined by RT-PCR amplification of 3 sub-genomic regions, followed by Sanger sequencing and phylogenetic analysis. RT-PCR amplification of viral RNA extracted from plasma was performed using the Qiagen OneStep RT-PCR kits (Qiagen, GmbH. Hilden, Germany) following the manufacturer’s protocol. A region of *gag* p24 (632 nucleotides in length) was amplified using primers p24-1F (5’AGYCAAAATTAYCCYATAGT3’) and p24-7R (5’CCCTGRCATGCTGTCATCA3’), a region of *pol* integrase (1009 nucleotides) amplified using primers poli5F (5’CACACAAAGGRATTGGAGGAAATG3’) and poli8R (5’TAGTGGGATGTGTAC TTCTGAAC3’), and a region of *env* gp41 (677 nucleotides) amplified using primers JH35 (5’TGARGGACAATTGGAGAARTGA3’) and JH38R (5’GGTGARTATCCCTKCCTAAC3’). Viral loads were determined using the RealTi*m*e HIV-1 assay following the manufacturer’s package insert (Abbott Molecular Inc., Des Plaines, IL). Specimen with high viral loads ranging from 4.45 to 5.90 log_10_ copies/mL and representing the diverse subtypes, rare CRFs, and URFs present in Cameroon were selected for NGS (Table 1).

**Table 1:** Phylogenetic classification and next-generation sequencing results for HIV-infected Cameroonian blood donors.

			HIV PCR/Sanger Sequencing								NGS			
No.	Sample ID^*a*^	HIVTiter^*b*^	*gag* p24	*pol* IN	*env* IDR	HIV Class.	HIV Read Count	HIV Read (%)	Cov (%)^*c*^	Reference Sequence	HIV Class.^*d*^	HPgV Reads	HPgV (%)	Cov (%)^*e*^
1	06CM-263-26	5.23	CRF11	CRF11	CRF11	CRF11	75,767	0.60	100	AF492624	CRF11	602,896	4.78	100
2	06CM-740-14	5.01	G	G	G	G	39,799	0.29	100	AF061642	G	629,689	4.58	100
3	06CM-876-14	5.38	D	D	D	D	36,919	0.21	100	K03454	**URF**	0	0	0
4	06CM-1130-39	4.91	CRF37	CRF37	CRF37	CRF37	23,055	0.16	100	AF004885	CRF37	0	0	0
5	06CM-1225-26	5.36	G	G	G	G	162,114	1.61	92	AF061642	G	0	0	0
6	06CM-1340-10	5.28	F2	F2	F2	F2	47,823	0.35	90	JX140673	F2	0	0	0
7	06CM-B460-1	5.53	CRF11	CRF11	CRF11	CRF11	86,590	0.81	82	AF492624	CRF11	18,251	0.17	87
8	07CM-46-10	5.68	A	A	A	A	32,475	0.61	100	AF004885	A	0	0	0
9	07CM-62-11	5.00	CRF11	CRF11	CRF11	CRF11	1,689	0.01	98	AF492624	CRF11	35,516	0.26	100
10	07CM-280-10	5.13	CRF22	A	CRF22	URF	5,903	0.04	100	AF004885	URF	250,766	1.72	100
11	07CM-419-33	5.55	CRF22/U	A/CRF02	CRF22	URF	381	0.003	78	AY371165	na	0	0	0
12	07CM-469-66	5.90	CRF36	CRF11	A/CRF02	URF	10,640	0.09	100	AF492624	URF	6,430	0.06	99
13	07CM-567-16	5.43	CRF22	CRF22/06	CRF02	URF	45,625	0.55	100	L39106	URF	0	0	0
14	07CM-640-14	5.09	G	G	G	G	17,045	0.10	96	AF061642	G	0	0	0
15	07CM-663-13	5.15	CRF22	CRF22/02	CRF22/36	URF	9,576	0.04	100	L39106	URF	0	0	0
16	07CM-920-49	5.36	CRF43	CRF43/U	CRF43	URF	108,797	0.62	100	AF061642	**G**	799,793	4.57	100
17	07CM-943-11	4.73	CRF22	CRF22	CRF22	CRF22	24,384	0.15	94	AY371165	CRF22	0	0	0
18	08CM-38-38	4.83	CRF22	CRF22	CRF22	CRF22	682	0.01	66	AY371165	na	0	0	0
19	08CM-228-10	4.94	CRF13	CRF13	CRF13	CRF13	38,043	0.27	100	AF460972	CRF13	0	0	0
20	08CM-669-39	5.17	CRF22	CRF22	CRF22	CRF22	5,334	0.33	92	AY371165	CRF22	0	0	0
21	08CM-789-10	5.47	G	G	CRF06	URF	23,226	0.14	100	L39106	**G**	0	0	0
22	08CM-833-62	5.58	CRF13	CRF13	CRF13	CRF13	54,772	1.17	100	AF460972	CRF13	237,565	5.07	100
23	08CM-867-10	5.10	H	H/A	URF	URF	2,196	0.01	100	AF004885	URF	0	0	0
24	08CM-886-24	4.86	A	A	A	A	10,371	0.21	100	AF004885	A	0	0	0
25	08CM-1252-11	4.83	CRF11	CRF11	CRF11	CRF11	1,724	0.01	100	L39106	**URF**	0	0	0
26	11CM-1156-26	4.48	CRF01	CRF01	CRF01	CRF01	11,168	0.09	100	AF004885	CRF01	0	0	0
27	11CM-B4043-15	5.30	CRF18	CRF18	CRF18	CRF18	377,128	4.51	100	AY586540	CRF18	0	0	0
28	11CM-CHU3903	4.61	CRF22	F2/CRF22	CRF22	URF	4,141	0.05	100	AY371165	URF	0	0	0
29	11CM-CHU2727	5.46	A	A	H	URF	6,151	0.07	100	AF004885	URF	18,018	0.21	100
30	11CM-CHU2801	4.47	CRF25	CRF25	CRF25	CRF25	140	0.002	53	DQ826726	na	0	0	0
31	02CM-A1575	4.45	CRF11	CRF11	CRF11	CRF11	6,451	0.04	100	L39106	CRF11	0	0	0
32	02CM-A1774	4.53	CRF11	CRF11	CRF11	CRF11	8,682	0.05	100	L39106	CRF11	0	0	0
33	04CM-260-50	5.11	CRF11	CRF11	CRF11	CRF11	6,511	0.06	100	L39106	CRF11	0	0	0
34	04CM-119-28	5.20	CRF11	CRF11	CRF11	CRF11	10,089	0.13	100	L39106	CRF11	0	0	0
35	04CM-1230-24	5.36	CRF11	CRF11	CRF11	CRF11	72,536	0.86	100	L39106	CRF11	0	0	0

^*a*^ Sample IDs are preceded by code denoting year of collection and country of origin (i.e., 02CM for 2002 in Cameroon).

^*b*^ Viral loads in log_10_ cps/ml.

^*c*^ HIV genome coverage.

^*d*^ NGS classifications in bold differ from RT-PCR classifications.

^*e*^ HPgV genome coverage.

### Pre-extraction filtering and DNase treatment

Plasma was thawed and spun at 4,000 x *g* for 10 minutes to pellet cell debris. Supernatants were passed through a 0.22 μm filter (Millipore, Billerica, MA) by spinning at 5000 x *g* for 5 minutes. For a 400 μl total volume, 331.2 μL of clarified plasma was combined with 20 μL Turbo DNase (Life Technologies, Carlsbad, CA, USA), 40 μL 10X Turbo Buffer, and 8.6 μL Baseline ZERO DNase (Epicentre, Madison, WI). This was incubated at 37°C or room temperature for 30 minutes with mixing by gentle vortexing after 15 minutes.

### RNA extraction

RNA was extracted from pre-treated plasma using the EZ1 Virus Mini Kit v2.0 protocol for serum on a Qiagen robot per manufacturer instructions, with the exception that 10 μl of 5 mg/ml linear acrylamide (Ambion/Life Technologies, Carlsbad, CA) was substituted for carrier RNA. Nucleic acid was eluted in 60 μl of Qiagen buffer AVE and either used immediately or stored at -70°C until use.

### cDNA NGS library preparation

Libraries were constructed from amplified cDNA using a modified TruSeq (Illumina, San Diego, CA) protocol as previously described [35–37]. Briefly, in Round A, RNA was reverse transcribed with MMLV SuperScript III Reverse Transcriptase (Invitrogen/Life Technologies, Carlsbad, CA) using Sol-PrimerA (5’-GTTTCCCACTGGAGGATA-N_9_-3’) that has a random 9-mer linked to a specific 17-mer containing the *BpmI* type IIs restriction enzyme site CTGGAG (Sol-Primer B, 5’-GTTTCCCACTGGAGGATA-3’), followed by second strand DNA synthesis with Sequenase (Affymetrix, Cleveland, OH). Reaction conditions for Round A were as follows: 1 μL of Sol-PrimerB (40 pmol/μl) was added to 11 μl of sample RNA, heated at 65°C for 5 minutes, then cooled at room temperature for 5 minutes. 8 μl of SuperScript Master Mix (4 μl 5X First-Strand Buffer, 2 μl 12.5 mM dNTP mix, 1 μl 0.1M DTT, 1 μl SS III RT) was then added and incubated at 42°C for 60 minutes. For second strand synthesis, single strand cDNA (20 μl) was denatured at 94°C for 2 minutes, returned to 10°C for 5 minutes, then 2.5 μl of Sequenase Mix #1 (1.5 μL 5X Sequenase Buffer, 0.775 μL ddH_2_O, 0.225 μl Sequenase enzyme) was added. Reactions were held at 37°C for 8 minutes, denatured again at 94°C for 2 minutes, returned to 10°C for 5 minutes, then 0.9 μL of Sequenase Mix #2 (0.675 μl Sequenase Dilution Buffer, 0.225 μl Sequenase Enzyme) was added. Incubations at 37°C for 8 minutes and 94°C for 2 minutes were repeated.

In Round B, Sol-PrimerB was used to amplify the randomly primed library. PCR products were purified, digested with *BpmI* to remove Sol-PrimerB, re-purified, and ligated to TruSeq adapters, followed by amplification with Illumina index-containing primers (Illumina). Round B reaction conditions were as follows: 10 μL of Round A -labelled cDNA was added to 40 μL of KlenTaq master mix per sample (5 μL 10X KlenTaq PCR buffer,1 μL 12.5 mM dNTP, 1 μL Sol-PrimerB (100 pmol/μl), 1 μL KlenTaq LA (Sigma-Aldrich, St Louis, MO), 32 μL ddH_2_O) and incubated as follows: 94°C for 2 minutes; 25 cycles of 94°C for 30 sec, 50°C for 45 sec, 72°C for 60 sec; 72°C for 5 minutes; hold at 10°C. Following amplification, 50 μl (1X) of Agencourt AMPure XP beads (Beckman Coulter, Brea CA) was added to Round B reaction mixture and incubated at room temperature for greater than 5 minutes. The beads were captured with a magnet and the supernatant was removed. Beads were then washed twice with 200 μL of 75% EtOH and air dried for 5 minutes. Libraries were eluted off the beads in 40 μl of water or elution buffer (Qiagen). For removal of Sol-PrimerB, Sol-PrimerB was cleaved with *BpmI* restriction enzyme (New England BioLabs, Ipswich, MA). Five μl NEB buffer 3, 5 μl 10xBSA, and 2 μl *BpmI* were added to 200 ng of Round B cDNA diluted in 38 μl of water and incubated at 37^°^C for 2 hr, followed by inactivation at 65^°^C for 20 minutes. Bead-based purification was repeated as described above and libraries eluted in 32 μl water or Qiagen EB buffer.

For end repair, ‘A’ addition, and Illumina adaptor ligation, 20 μl of mastermix (5 μl 10X T4 ligase buffer, 1 μl T4 DNA polymerase, 1 μl of T4 Polynucleotide Kinase, 0.5 μl Klenow, 10.5 μl water) was added to 30 μl of eluted library. Reactions were incubated at 20^°^C for 30 minutes, bead-based purification was repeated, and libraries were eluted from beads in 17 μl water or Qiagen EB buffer. Ten μl of mastermix (2.5 μl 10X Buffer 2, 5 μl 1mM dATP, 1 μl Klenow exo-, 1.5 μl water) was added to 15 μl of eluted library. Reactions were incubated at 37^°^C for 30 minutes, Agencourt AMPure XP beads (1.0X ratio = 25 μl beads) purifications were repeated, and libraries were eluted in 17 μl water or EB. 10 μl of mastermix (2.5 μl 10X T4 ligase buffer, 1 μl PE adapter oligo mix (5’-ACACTCTTTCCCTACACGACGCTCTTC CGATCT-3’ and 5’-GATCGGAAGAGCACACGTCT-3’, 1 μl T4 DNA ligase, 5.5 μl water) was added to 15 μl of end-repaired cDNA and incubated at room temperature overnight. Ligated material was brought up to 50 μl with water, Agencourt AMPure XP beads (1.0X ratio = 50 μl beads) purifications were repeated, and eluted in 25 μl of water.

For PCR-based index addition and library purification, 1 μL-10 μL of sample was used as input with 10 μL 5X Phusion buffer, 1 μL 12.5 mM dNTP, 2 μL IDT-made index (TruSeq Universal Adapter: 5’-AATGATACGGCGACCACCGAGATCTACACTCTTTCCCTACAC GACGCTCTTCCGATCT-3’), 1 μL IDT-made InPE1.0 (3’ portion of TruSeq Indexed Adapter: 5’-GTGACTGGAGTTCAGACGTGTGCTCTTCCGATCT-3’), 1 μL IDT-made InPE2.0 (5’ portion of TruSeq Indexed Adapter containing a 6-base barcode sequence: 5’-CAAGCAGAAGACGGCATACGAGATNNNNNNGTGACTGGAGTTC-3’) and 1 μL of Phusion enzyme (Fisher Scientific, Tewksbury, MA), with water amounts adjusted to have a 50 μL total volume. Libraries were amplified as follows: 98^°^C for 30 sec; 17 cycles of 98^°^C for 15 sec, 65^°^C for 30 sec, 72^°^C for 30 sec; 72^°^C for 5 minutes; hold at 10^°^C. Libraries were purified as above with AMPure beads, and eluted in 30 μL. Library size was determined using a High Sensitivity DNA kit on an Agilent BioAnalyzer 2100 instrument (Agilent, Santa Clara, CA) and concentration was measured using a KAPA Library Quantification Kit (Kapa Biosystems, Woburn, MA).

### NGS and data analysis

Libraries were each diluted to 1 nM. Five libraries were multiplexed, denatured in 0.1N NaOH for 5 minutes, and then diluted to 20 pM in Illumina buffer HT1. PhiX internal control was added at a 1% final concentration to 10 pM libraries. Sequencing was performed on a MiSeq instrument using a 500 cycle MiSeq Reagent Kit v2 (Illumina). Barcodes were parsed on the MiSeq instrument and filtered for Q-scores above 30. Paired-end reads 1 and 2 were merged and aligned to an HIV-1 reference sequence using CLC Genomics Workbench 6.02 software (CLC bio/Qiagen, Aarhus, Denmark). The reference sequence from the GenBank database was selected based on the classification of *gag*, *pol*, and *env* sub-genomic sequences obtained from each specimen (Table 1). The NGS reads were then realigned to the consensus through repeated iterations to obtain a final consensus sequence. Open reading frames in each consensus HIV-1 genomic sequence were verified, edited where required, and annotated. For HPgV, NGS reads were aligned to reference sequence NC_001710. GenBank accession numbers for full length HIV sequences are: KP718914 (263–26), KP718915 (740–14), KP718916 (876–14), KP718917 (1130–39), KP718918 (46–10), KP718919 (280–10), KP718920 (469–66), KP718921 (567–16), KP718922 (663–13), KP718923 (920–49), KP718924 (228–10), KP718925 (789–10), KP718926 (833–62), KP718927 (867–10), KP718928 (886–24), KP718929 (1252–11), KP718930 (1156–26), KP718931 (B4043-15), KP718932 (CHU3903), KP718933 (CHU2727), KP718934 (A1575), KP718935 (A1774), KP718936 (260–50), KP718937 (119–28), KP718938 (1230–24), and for HPgV sequences are: KP710598 (263–26), KP710599 (740–14), KP710600 (62–11), KP710601 (280–10), KP710602 (469–66), KP710603 (920–49), KP710604 (833–62), KP710605 (CHU2727), KP710606 (8013815).

### Phylogenetic Analysis

To classify the HIV -1 strains, final genomic consensus sequences were aligned with HIV-1 group M reference sequences [2] using the CLUSTALW method (MegAlign, Lasergene version v11 DNASTAR Inc., Madison, WI). Alignments were converted into PHYLIP format using ForCon (version 1.0 for Windows; J. Raes, University of Ghent, Belgium) and gap-stripped using BioEdit Sequence Alignment Editor (version 5.0.9, Tom Hall, North Carolina State University, Raleigh, North Carolina). Phylogenetic analysis was performed with the PHYLIP software package (version 3.5c; J. Felsenstein, University of Washington, Seattle, WA). Evolutionary distances were estimated with DNADIST (Kimura two-parameter method) and phylogenetic relationships were determined by NEIGHBOR (neighbor-joining method). Branch reproducibility of trees was evaluated using SEQBOOT (100 replicates) and CONSENSE. Programs were run with default parameters. Trees were constructed using TreeExplorer (version 2.12; Dr. Koichiro Tamura of Tokyo Metropolitan University, Tokyo, Japan; [38]).

For each CRF, bootstrap analysis was performed and phylogenetic trees were constructed for each sub-fragment (data not shown) to verify the predicted recombinant structure. Recombination analysis was performed using SIMPLOT (version 3.5.1; S. Ray, Johns Hopkins University, Baltimore, MD; [39]). Viral sequences were individually evaluated in SIMPLOT for evidence of recombination relative to subtypes and CRFs. If SIMPLOT indicated evidence of recombination, Bootscan and Findsite were performed; recombination was confirmed by constructing phylogenetic trees for each sub-fragment. The 8 Cameroonian HPgV strains in the present study (either the complete genomic sequences or the 5’UTR sequences) were aligned with 47 HPgV reference sequences in the database and analyzed in the same manner as described above for HIV.

## Results

### Next Generation Sequencing for Viral Characterization

Plasma specimens were obtained from asymptomatic HIV-1 infected blood donors from 2002–2011 in the Cameroonian cities of Yaoundé and Douala. The initial classification of HIV strains was based on RT-PCR amplification of three genome sub-regions (*gag* p24; *pol* integrase, and *env* gp41), followed by traditional Sanger sequencing and phylogenetic analysis. A panel of 35 specimens harboring a variety of strains, including CRFs for which few reference sequences exist and potential URFs not previously reported, as well as different subtypes present in the population, was selected for complete genomic characterization by NGS (Table 1). To maximize the likelihood of obtaining full-length genomes, specimens were chosen with viral loads greater than 4.4 log_10_ copies/ml (Table 1).

**Fig 1:**
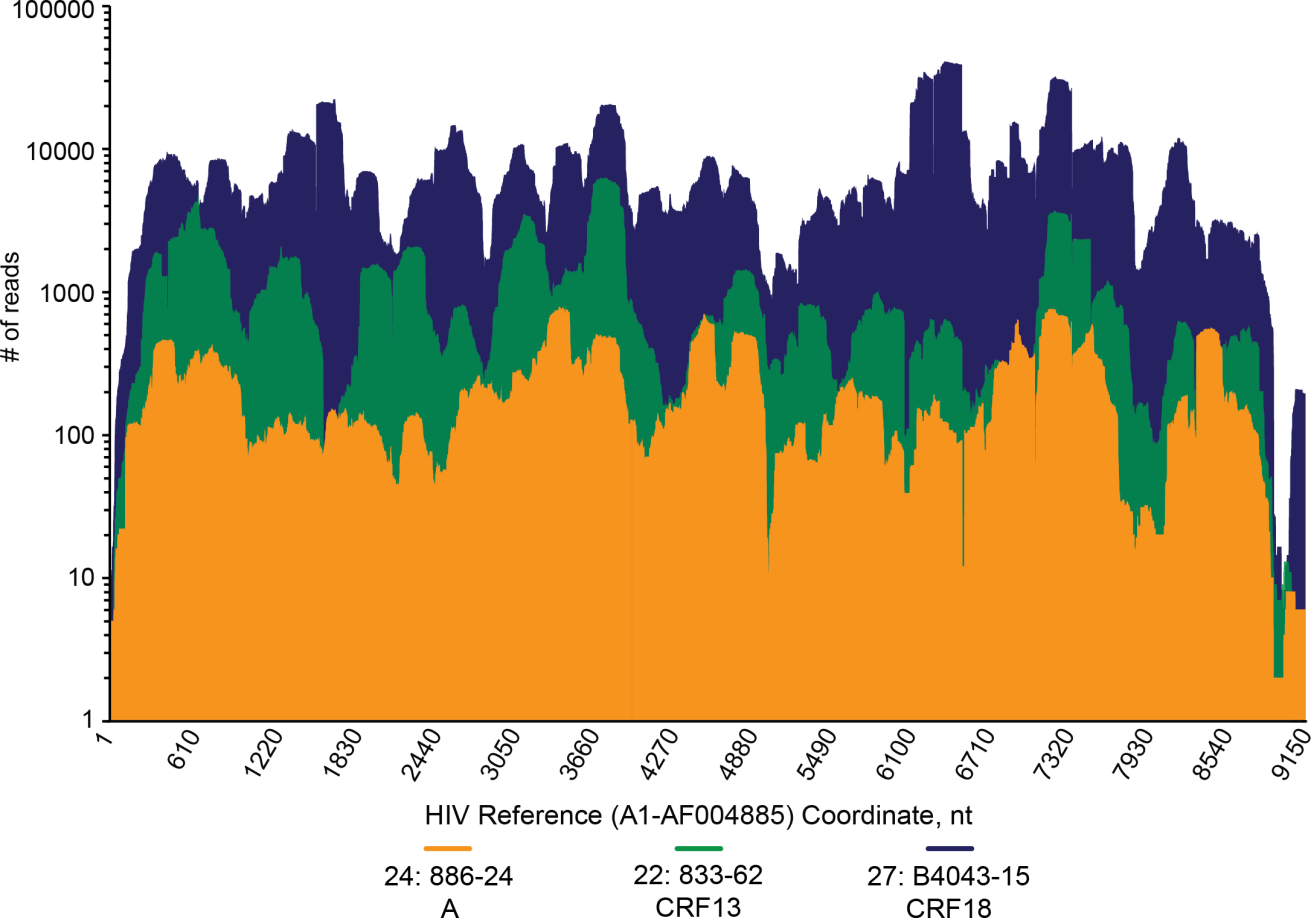
HIV genome coverage is uniform and complete but varies in sequence depth. Three representative specimens sequenced by NGS with a wide range in percentage of HIV reads were selected and aligned to the A1-AF004885 reference genome to demonstrate the uniformity of genome coverage regardless of read depth. Coverage is expressed as number of reads at each nucleotide position along the length of the HIV genome. Strain/mean read number: 833-62/1085, green; 886-24/223, orange; B4043-15/7939, blue.

To reduce background from human host genomic sequences, plasma specimens were pre-treated with a cocktail of nucleases and filtered prior to NGS library preparation and sequencing [40]. An average of 11.8 ± 4.6 million high-quality reads per specimen were obtained by 2x250 base pair (bp) paired-end sequencing on a MiSeq instrument. Despite the uniformity in input virus titer, the number of reads aligning to a reference HIV genome varied greatly from one library to the next. The number of HIV reads ranged from a low of 140 to a high of 377,128, with a median of 11,168 reads per specimen (Table 1). Even after pre-treatment with nucleases to reduce human nucleic acid background, HIV reads still represented a small proportion of the total sequences obtained from each specimen (0.002–4.51% of reads with a median of 0.14%). However, greater than 90% genome coverage was achieved for 31 (88%) of the 35 specimens, with average sequence depth ranging from 37- to 8,274-fold, permitting full-length assembly of the viral genome for 25 (71%) specimens. For libraries yielding full-length genomes, consensus sequences were reproducible from run to run (≥ 99% identity) and agreed with population (Sanger) sequence results. Coverage depth was relatively uniform with no particular bias towards or against any region, with the exception of the 5’ and 3’ ends (Fig 1). The same is true for libraries with gaps in genome coverage (S1–S7 Figs); regions with few to no reads varied randomly from one library to the next. Despite residual gaps in coverage, phylogenetic trees derived from gap-stripped alignments of these 7 incomplete genomes (≥80% coverage) showed that each branched with 100% bootstrap values with the subtype expected from sub-genomic sequences (S1–S7 Figs).

**Fig 2:**
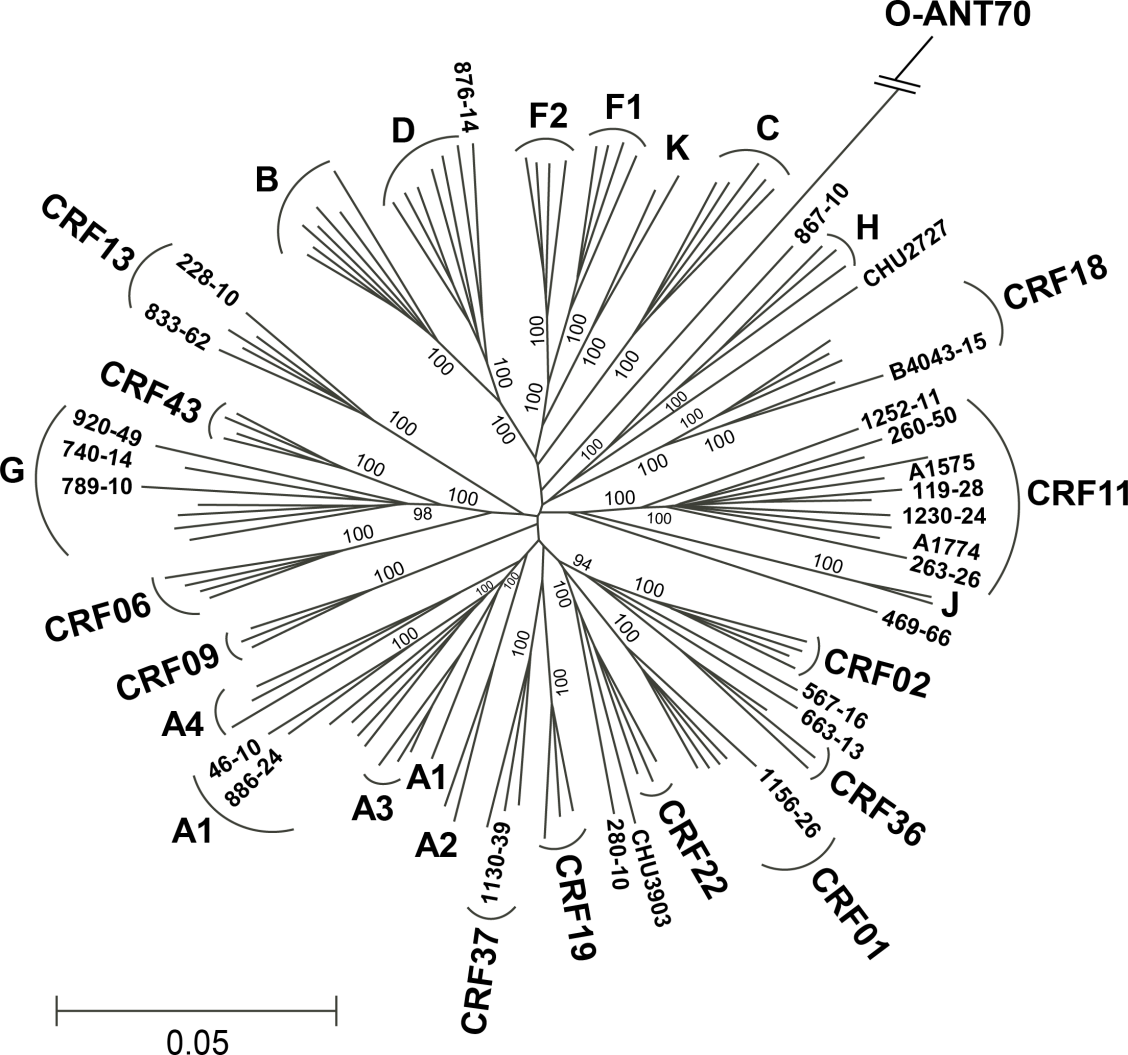
Phylogeny of full length genomes obtained by NGS illustrates HIV diversity in Cameroon. A phylogenetic tree of 92 HIV-1 complete genome reference sequences and 25 Cameroonian sequences was constructed from a 7387 bp gap-stripped alignment with bootstrap values indicated at each branch. Group O strain ANT70 was used as the outgroup and the genetic distance scale is indicated.

**Fig 3:**
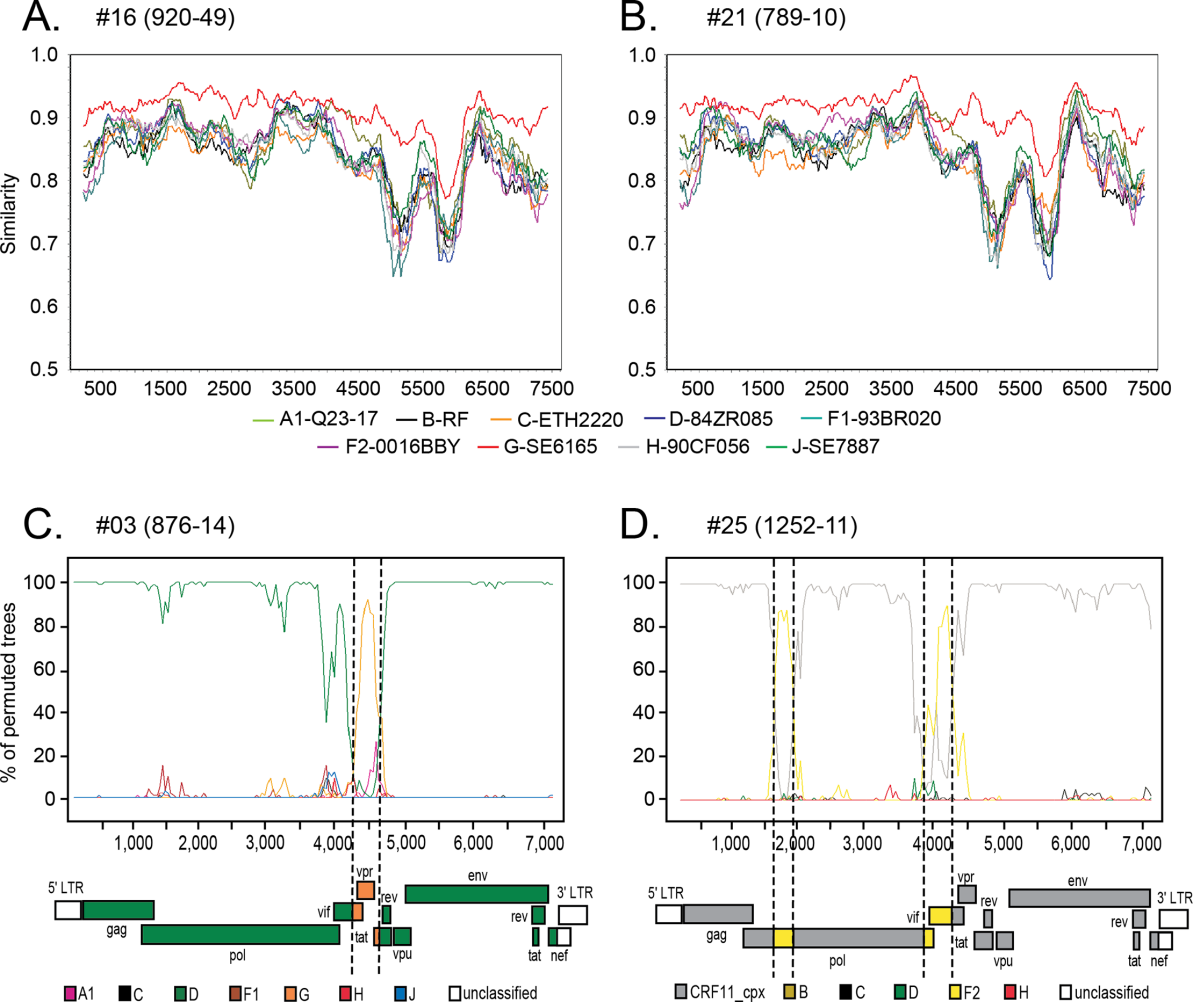
Full genome bootscanning reveals the true extent of recombination. Sequences 920–49 **(A)** and 789–10 **(B)** were evaluated in SIMPLOT against pure subtype reference sequences and both found to be subtype G throughout the genome (red line). Specimen 876–14 **(C)** and 1252–11 **(D)** were subjected to SIMPLOT bootscanning analysis; the vertical dashed lines indicate recombination breakpoints. The genomic structure is diagramed below each bootscan plot. Bootscan and SIMPLOT analysis was performed using a window of 400 base pairs and 20 base pair step.

Phylogenetic analysis of an alignment of the 25 full-length HIV-1 genomes obtained by assembly of the NGS reads and 92 references representing group M subtypes A-D, F-H, J-K and selected CRFs was performed using group O strain ANT70 as the outgroup (Fig 2, Table 1). Separate phylogenetic trees were also constructed for 7 partially sequenced genomes (82–98% genome coverage; S1–S7 Figs) while 3 samples (419–33, 38–38, and CHU2801) having ≤ 78% coverage were not analyzed further. Comparison of strain classification based on a previous algorithm derived from the sequences of the *gag*, *pol*, and *env* sub-regions versus NGS and genome assembly showed that they were concordant in 31 of 35 (89%) cases (Table 1) [21]. Recombination analysis revealed that two strains, 789–10 and 920–49, originally classified as URFs, were in fact pure subtype G (Figs 2, 3A and 3B). The *env* sequence for strain 789–10 grouped strongly with CRF06, which is comprised of subtypes G and J in this region. However, subtype J sequences were not found to be present in the full 789–10 genome sequence (Fig 3A). Similarly, strain 920–49 grouped strongly with CRF43 in *gag* and *env*, designated as G in these regions, but recombination analysis confirmed the subtype G classification across the whole length of the genome, indicating that strain 920–49 was not a recombinant (Fig 3B). In addition, for two strains, 876–14 and 1252–11, NGS allowed identification of recombinants that were not revealed by sub-genomic sequences; these two strains showed basal branches within their respective phylogenetic clusters (Figs 2, 3C and 3D). Strain 876–14, originally designated as subtype D, was found to contain subtype G sequences in the *vif/vpr* region (Fig 3C). Similarly, strain 1252–11, originally designated as subtype CRF11, was found to contain subtype F2 regions in *pol* RT and *vif* (Fig 3D).

### Molecular Characterization of Low Prevalence Circulating Recombinants

**Fig 4:**
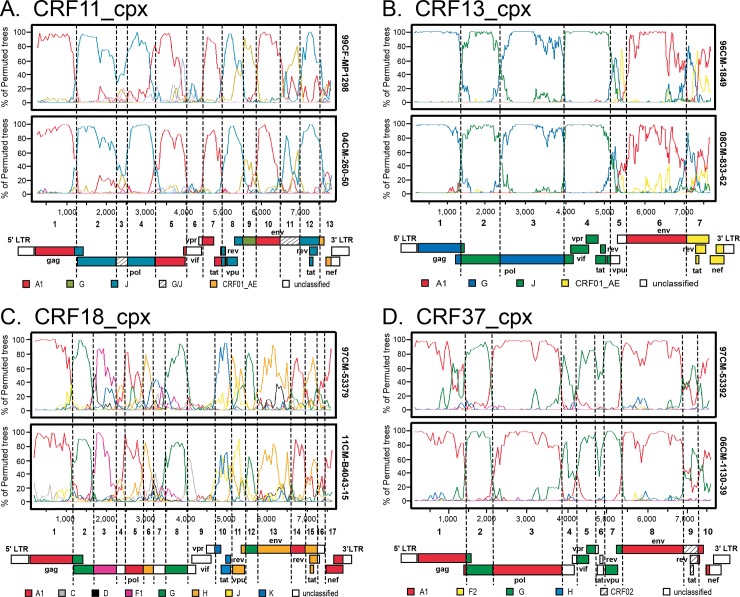
Breakpoint analysis for rare, complex recombinants endemic to Cameroon. Bootscan plots are shown for **(A)** CRF11_cpx, **(B)** CRF13_cpx, **(C)** CRF18_cpx and **(D)** CRF37_cpx isolates. In each panel the profile for a reference strain is shown on top and a representative new strain is on the bottom. Vertical dashed lines indicate recombination breakpoints determined by Find Site; genome structure is diagramed below each plot. Bootscan analysis was performed using a window of 400 base pairs and 20 base pair step.

Limited full-genome data are available for rare CRFs found in Cameroon [41], [42, 43], [44] [45], prompting us to focus on these recombinant strains. Six full-length HIV genomes derived from putative CRF11_cpx strains grouped at high confidence (bootstrap value 100%) with the CRF11_cpx reference strains in a well-defined monophyletic cluster (Fig 2). The pattern of recombination in each of the 6 genomes was identical to that in CRF11 reference strain 95CM-1816 [41] (Fig 4A).

CRF13_cpx is another complex recombinant endemic to central-west Africa, comprised of subtypes A1, G, J, and CRF01_AE sequences. The genomic sequences of 228–10 and 833–62 clustered with CRF13_cpx reference sequences (Fig 2), and share the identical genomic structure by recombination analysis (Fig 4B) [42, 43]. As in the CRF13_cpx reference strains, HIV strains 228–10 and 833–62 contained 10 and 7 amino acid insertions, respectively in *gag* p6 [42, 43].

Additional low frequency CRFs were analyzed in the same manner (Fig 4C and 4D). B4043-15 is a CRF18_cpx, a highly complex recombinant consisting of 15 fragments derived from several subtypes (A, F, G, H, K and unclassified). The mosaic composition of B4043-15 strongly resembles CRF18 reference strains from both Cuba and Cameroon (Fig 4C). Strain 1130–39 grouped tightly with the other CRF37_cpx references from Cameroon (Fig 2) and recombination analysis confirmed this designation; 1130–39 is a complex recombinant comprised of subtypes A and G and unclassified regions with 9 breakpoints (Fig 4D).

### Full Genome Sequence Analysis of 9 Unique Recombinant Forms

**Fig 5:**
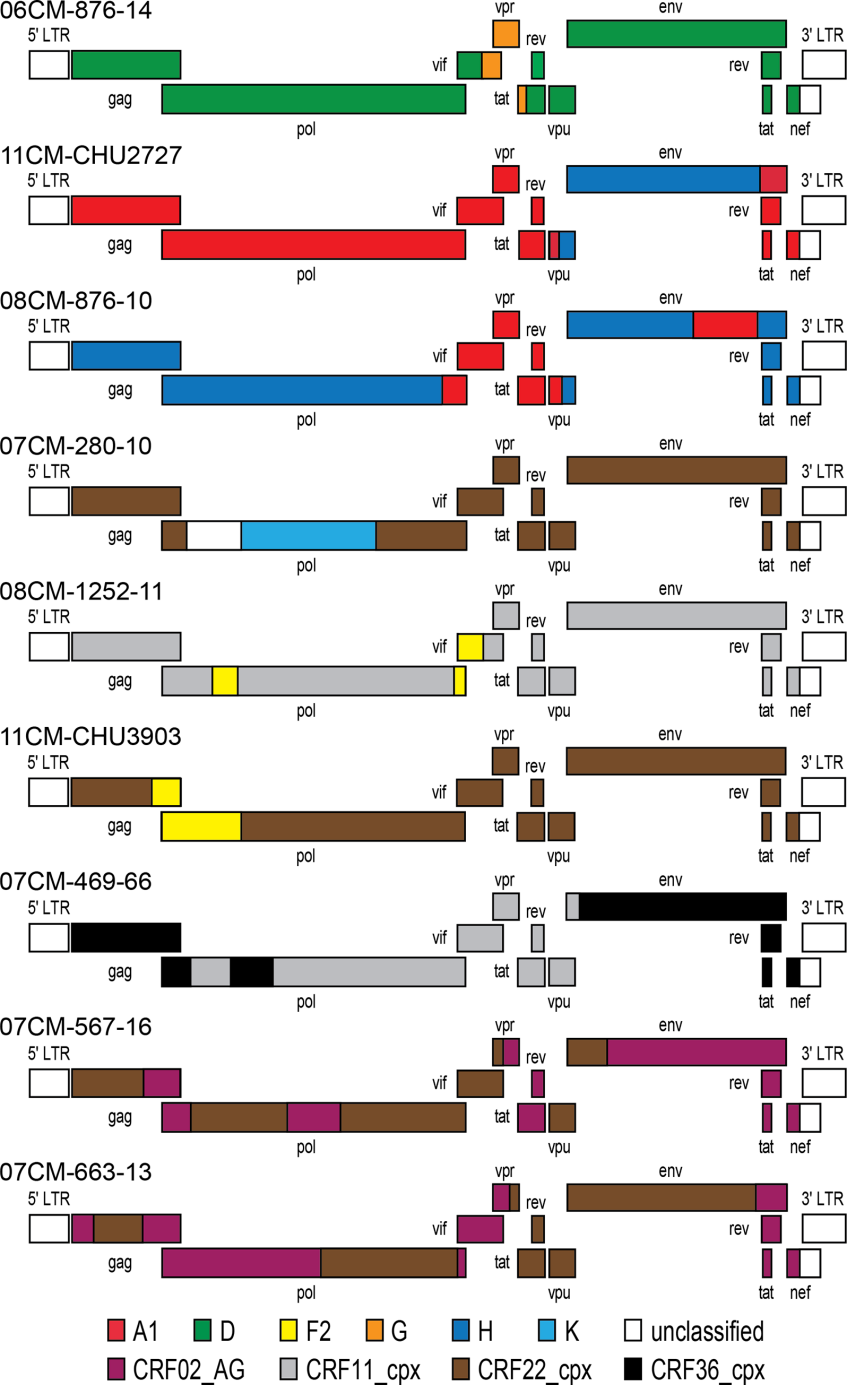
Genetic organization of unique recombinant forms obtained by NGS. Nine unique recombinants are shown with the legend at the bottom indicating the classification of each sub-genomic fragment. Genomic coordinates for each recombination breakpoint are described in detail in the Supplemental information.

Nine unique recombinant forms (URFs) were identified (Fig 5) whose phylogenetic trees of sub-fragments are shown in S8–S16 Figs. The first set of three URFs represents recombination events between pure subtypes. Strain 876–14, previously shown in Fig 3C, is subtype D interrupted by a short stretch of subtype G sequence (nt 5342–5887) from *vif* to *tat*. CHU2727 is subtype A, interrupted by a 2.3 kb stretch of subtype H sequence (nt 6091–8335) from *vpu* to the 3’ half of *gp41*. Strain 867–10 consists of 5 fragments that alternate between subtypes H and A sequence.

The next three URFs are the result of recombination between pure subtypes and a CRF. Strain 280–10 is predominantly CRF22_01A1, interrupted by unclassified sequence (nt 2424–2903) from protease to the beginning of RT, and subtype K sequence (nt 2904–4235) from the 3’ portion of RT to the beginning of integrase. 1252–11 is a CRF11-cpx that contains two short segments of F2 sequence in the 5’ portion of RT (nt 2584–2863) and the 3’ end of integrase through *vif* (nt 4872–5401). Strain CHU3903 is all CRF22_01A1 sequence except for 1 kb of subtype F2 (nt 1982–2958) which spans the 3’ half of *gag* through the 5’ portion of *pol* RT.

The third set of three URFs contain sequences from two different CRFs. 469–66 consists of 5 fragments that alternate between CRF36_cpx and CRF11_cpx sequences. The next two strains (567–16 and 663–13) consist of alternating stretches of CRF02_AG and CRF22_01A1 sequences but do not have the same recombination breakpoints. Notably, while the majority of these specimens were correctly classified as URFs based on sequences from three sub-regions, the benefit of full genome coverage allowed the recombination breakpoints to be precisely identified.

### Detection of HPgV/GBV-C co-infection

The use of random priming for library generation allowed us to interrogate the specimen NGS data for the presence of additional viral agents. Of particular interest is the human pegivirus (HPgV, family *Flaviviridae*), also known as GB virus-C (GBV-C). NGS data were aligned to HPgV reference genome NC_001710; HPgV reads were detected in 9 of 35 (26%) specimens. Seven specimens yielded complete genome sequences and one with 99% of the genome. For most of these specimens, the percentage of HPgV reads (0.06–5.07%) far exceeded that obtained for HIV (Table 1). No correlation was found between HIV subtype and co-infection with HPgV.

**Fig 6:**
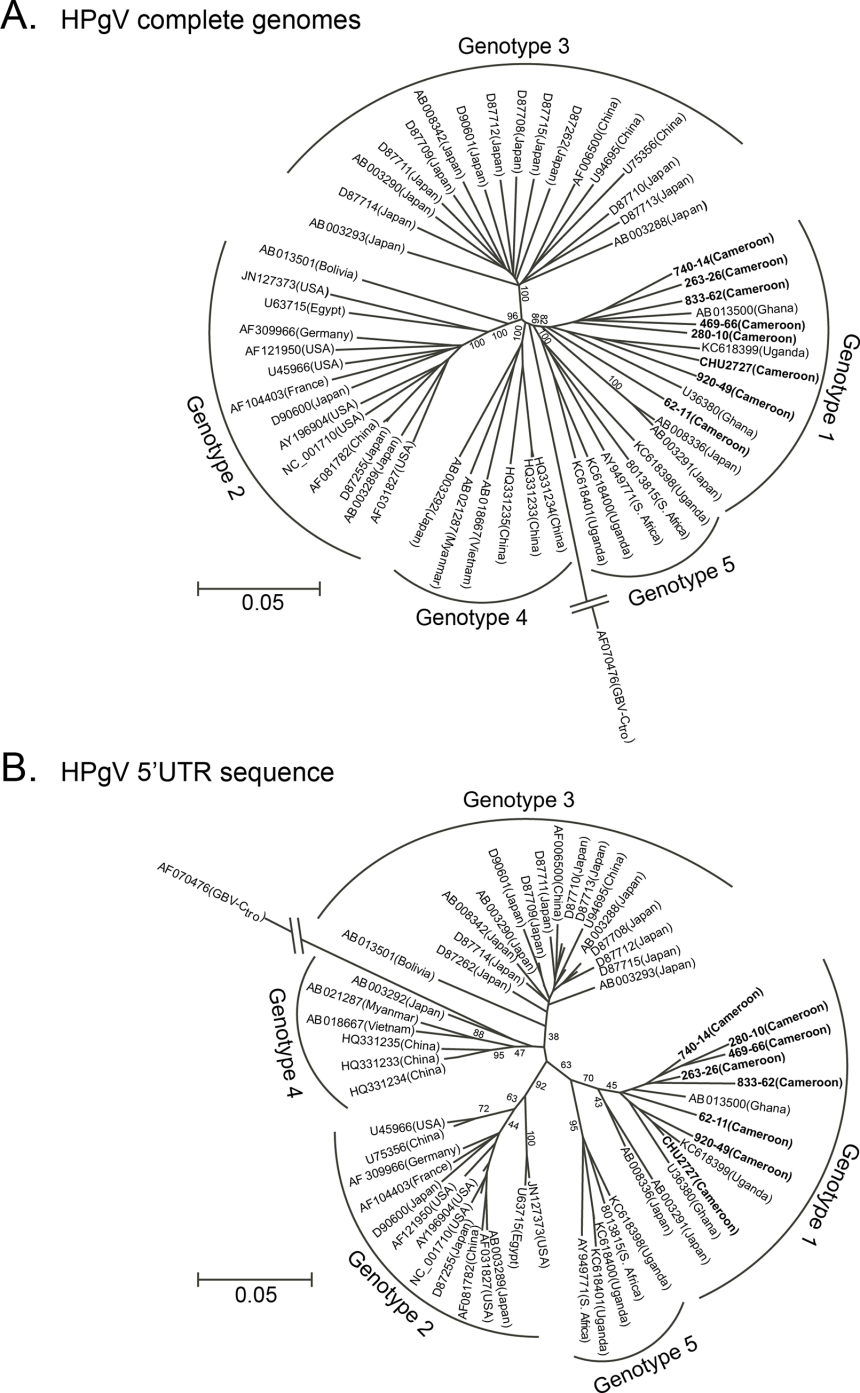
Phylogenetic trees of HPgV indicate Cameroon sequences group with genotype 1. Phylogenetic trees of 56 HPgV **(A)** complete genome sequences (8851 nt after degapping) and **(B)** 5’UTR sequences (366 nt) of 8 Cameroonian sequenced by NGS in this study, were constructed with bootstrap values indicated at each branch. GBV-C*tro* was used as the outgroup and the genetic distance scale is indicated. References are labeled individually with accession number and country of origin; Cameroonian sequences are in bold text.

The 8 Cameroonian HPgV sequences were aligned with a total of 46 HPgV reference sequences including genotypes 1–5, and a chimpanzee isolate (GBV-Ctro) as the outgroup. Phylogenetic trees were constructed based on the full genome alignment (Fig 6A). The Cameroonian genomes were found to cluster within the genotype 1 branch consistent with their African origin. Since the most recent common ancestor occupied a basal position on the genotype 1 branch and the sequences were separated from each other by relatively long branch lengths, the genotype 1 classification could be confirmed by phylogenetic analysis of the 5’UTR sequence alignment (Fig 6B) [46]. No evidence of recombination was observed (data not shown). Sequence identity between these new isolates varied from 89–94%, confirming that each strain was derived from a unique specimen.

## Discussion

The continuing diversification and global dynamics of HIV groups, subtypes and recombinants, as well as the emergence of new strains make it imperative that surveillance of viral diversity be conducted to monitor the dynamic HIV epidemic. While traditional methods (i.e. peptide-based serotyping, heteroduplex mobility assays, and Sanger sequencing of sub-genomic regions) have provided useful data, the metagenomic NGS approach used here enabled full-genome sequencing of HIV-1 with unequivocal strain classification directly from primary clinical samples, with clear applications for routine viral surveillance. Even for specimens with incomplete coverage (e.g. 80–95%), enough sequence information was obtained to accurately classify strains. The depth of genome coverage achieved by NGS instills confidence in the consensus sequence generated, while analysis of individual reads affords the potential to identify dual infections, low frequency variants or quasispecies, and the evolution of intrahost populations [47–51]. Our analysis did not reveal infection with more than one HIV strain in any given patient, and only one individual possessed a common drug resistance mutation (data not shown). The ability to multiplex specimens also increases the throughput of NGS and lowers the per-patient cost of sequencing.

Given the diversity of HIV, we chose to generate libraries using random primers. This approach resulted in the assembly of complete (71%) or nearly complete (≥90% coverage) genomes for highly diverse HIV-1 strains, including subtypes A, F2, and G, CRFs CRF01_AE, CRF11_cpx, CRF13_cpx, CRF18_cpx, CRF22_01A1 and CRF37_cpx, and URFs. The depths of genome coverage obtained here are higher than two other recent reports using NGS for viral screening in blood [52, 53], and may be potentially be attributed to higher input viral titers. Despite nuclease pre-treatment, removal of host background was far from complete. Coupled to potential biases introduced during cDNA library amplification, host background likely contributed to the variability in % HIV reads and coverage depth observed for specimens with equivalent viral loads (Table 1). For example, 876–14 and 1225–26 had comparable viral loads of 5.38 and 5.36 log_10_ copies/ml, respectively, yet % HIV reads and genome coverage were 0.21% and 1.61%, and 100% and 92%, respectively. Similarly, CHU2810 and A1575 had viral loads of 4.47 and 4.45 log_10_ copies/mL, yet % HIV reads of 0.002% and 0.04%, and genome coverage of 53% and 100%, respectively. The intent of this study was to establish the feasibility of metagenomic NGS. For this reason, samples with high viral loads (>4.45 logs) were selected. We did not seek to address the sensitivity of the method at lower input viral titers. Additional optimization of the protocol using host depletion or probe enrichment strategies may be warranted to further increase the percentage of viral reads, thereby enhancing sensitivity and limits of detection [40].

With Cameroon at the epicenter of the HIV epidemic, our data contribute a number of full-genome recombinant HIV-1 sequences to the GenBank database. Reliance on partial genome PCR characterization for surveillance is likely to underestimate the true extent of HIV diversity. Indeed, in the current study, two unique recombinants were identified that would otherwise have been categorized as a pure subtype D (876–14) or CRF11 (1252–11), had it not been for the complete genome sequence. Longer contiguous sequences also facilitate more accurate phylogenetic classification; a segment of strain 789–10 was misclassified as CRF06 based on 677 nucleotide region of *env*, and strain 920–49 was misclassified as CRF43 based on short segments of *gag* and *env*. The availability of whole-genome sequencing for viral surveillance enables rapid determination of whether new strains may be circulating in Cameroon. It bears mentioning that all subtypes and CRFs found in these URFs are endemic to the region. Indeed, many of the observed sites of recombination (Figs 3 and 5) were found in known genomic hotspots in the *vif/vpr/vpu/tat* and *pol (RT)* regions [54, 55].

NGS with random-primed libraries has been used successfully to characterize the virome of clinical specimens [16, 36, 56], diagnose infections [57], and discover novel viruses [58, 59]. To assess the ability of NGS to identify co-infections with other viruses, we searched for HPgV sequences in the NGS data. HPgV sequences were detected in 9 of 35 (26%) specimen libraries, with 8 yielding at least 99% of the viral genome. HPgV has a worldwide distribution and is transmitted sexually, parenterally and by mother-to-child transmission, and as a consequence HIV patients are often co-infected [60]. The high rate (25%) of HPgV nucleic acid detection in our specimens is notable, given the documented lower prevalence of ~16% RNA positives in HIV-infected patients (4% in healthy individuals) ([19] and references therein).

HPgV genotypes exhibit consistent geographical clustering, with US and European strains exclusively genotype 2, and genotypes 3 (Japan and China) and 4 (Vietnam, Japan, and China) found primarily in Asia. Recently, complete genomes from Uganda in east Africa were assigned to either genotype 1 or to genotype 5, previously considered exclusive to South Africa [61]. In the present study, the 8 Cameroonian HPgV genomes clustered within genotype 1 along with 2 isolates from Ghana and 1 from Uganda, and more distant from 2 Japanese strains putatively assigned to genotype 6 [61]. The 5'UTR phylogenetic analysis supports the whole genome-based classification of the Cameroonian GBV-C strains as genotype 1.

NGS has ushered in a new era of discovery, as well as new challenges [62, 63]. Applications of this technique are accelerating pathogen discovery [14, 64, 65] and promise to transform clinical diagnostics [57] as NGS-based assays begin to move into the clinical laboratory. We propose here that NGS has the potential to be applied as a routine yet powerful approach for surveillance of viral diversity, as well as a tool to identify co-infections such as from HPgV. It is anticipated that these technological advances will lead to a more thorough understanding of the HIV epidemic and underlying sequence diversity.

## Supporting Information

S1 Information Inventory and genome structures of URFs. (PDF)

S1 Fig NGS coverage and phylogenetic classification of 06CM-1225-26. (TIF)

S2 Fig NGS coverage and phylogenetic classification of 07CM-640-14. (TIF)

S3 Fig NGS coverage and phylogenetic classification of 06CM-1340-10. (TIF)

S4 Fig NGS coverage and phylogenetic classification of 06CM-B460-1. (TIF)

S5 Fig NGS coverage and phylogenetic classification of 07CM-62-11. (TIF)

S6 Fig NGS coverage and phylogenetic classification of 07CM-943-11. (TIF)

S7 Fig NGS coverage and phylogenetic classification of 08-CM-669-39. (TIF)

S8 Fig Bootscanning and sub-fragment trees for URF_06CM-876-14. (TIF)

S9 Fig Bootscanning and sub-fragment trees for URF_07CM-280-10. (TIF)

S10 Fig Bootscanning and sub-fragment trees for URF_07CM-469-66. (PDF)

S11 Fig Bootscanning and sub-fragment trees for URF_07CM-567-16. (PDF)

S12 Fig Bootscanning and sub-fragment trees for URF_07CM-663-13. (PDF)

S13 Fig Bootscanning and sub-fragment trees for URF_08CM-867-10. (PDF)

S14 Fig Bootscanning and sub-fragment trees for URF_08CM-1252-11. (PDF)

S15 Fig Bootscanning and sub-fragment trees for URF_11CM-CHU3903. (PDF)

S16 Fig Bootscanning and sub-fragment trees for URF_11CM-CHU2727. (PDF)

S1 Table Cameroonian blood donor demographic data. (PDF)
