# Supplementary material for: Utility of Metagenomic Next-Generation Sequencing for Characterization of HIV and Human Pegivirus Diversity
Source: PLoS One. 2015 Nov 23;10(11):e0141723. doi: 10.1371/journal.pone.0141723 (PMC4658132; doi:10.1371/journal.pone.0141723)
Supplement: S1 Information — (PDF) [file pone.0141723.s001.pdf]

Online Supplement Inventory

Utility of Unbiased Next-Generation Sequencing for Characterization of HIV Diversity

Ka-Cheung Luk, Michael G Berg, Samia N Naccache, Beniwende Kabre, Scot Federman, Dora Mbanya, Lazare Kaptué, Charles Y Chiu, Catherine A Brennan and John Hackett Jr.

Supplemental Table S1. Cameroonian donor demographic data.

Supplemental Figures S1-S7 Coverage maps, NGS statistics and phylogenetic trees for specimens with partial genome coverage.

Genome structures of unique recombinant forms

Supplemental Figures S8-S16. Bootscanning and phylogenetic trees for unique recombinant forms.

Genomic Structures of the URF Recombinants
(numbering according to HXB2 complete genome, K03455)

<u>Supplemental Figure S8: 06CM-876-14</u>
Fragment 1 (nt 827-5341, subtype D) includes *gag*, *pol*, and the 5' half of *vif*.

Fragment 2 (nt 5342-5887, subtype G) includes the 3' half of *vif*, *vpr*, and the beginning of *tat*.

Fragment 3 (nt 5888-9012, subtype D) includes the 3' portion of *tat*, *rev*, *vpu*, *env*, the second exons of *tat* and *rev*, and the 5' one-third of *nef*.

<u>Supplemental Figure S9: 07CM-280-10</u>
Fragment 1 (nt 827-2423, CRF22_01A1) includes *gag* and the beginning of the *pol* protease.

Fragment 2 (nt 2424-2903, unclassified) includes the 3' portion of protease and the beginning of RT.

Fragment 3 (nt 2904-4235, subtype K) includes the 3' portion of RT and the beginning of *int*.

Fragment 4 (nt 4236-9012, CRF22_01A1) includes the 3' portion of *int*, *vif*, *vpr*, *tat*, *rev*, *vpu*, *env*, the second exons of *tat* and *rev*, and the 5' one-third of *nef*.

<u>Supplemental Figure S10 07CM-469-66</u>
Fragment 1 (nt 827-2463, CRF36_cpx) includes *gag* and the 5' half of the *pol* protease.

Fragment 2 (nt 2464-2843, CRF11_cpx) includes the 3' half of protease and the beginning of RT.

Fragment 3 (nt 2844-3232, CRF36_cpx) includes most of the 5' half of RT.

Fragment 4 (nt 3233-6349, CRF11_cpx) includes the 3' half of RT, *int*, *vif*, *vpr*, *tat*, *rev*, *vpu*, and the beginning of *env* gp120.

Fragment 5 (nt 6350-9012, CRF36_cpx) includes the 3' portion of *env* gp120, gp41, the second exons of *tat* and *rev*, and the 5' one-third of *nef*.

<u>Supplemental Figure S11: 07CM-567-16</u>
Fragment 1 (nt 827-1879, CRF22_01A1) includes *gag* p17 and p24.

Fragment 2 (nt 1880-2463, CRF02_AG) includes *gag* p2, p7, p6, and the 5' portion of the *pol* protease.

Fragment 3 (nt 2464-3393, CRF22_01A1) includes the 3' portion of protease and the 5' half of RT.

Fragment 4 (nt 3394-3875, CRF02_AG) includes most of the 3' half of RT.

Fragment 5 (nt 3876-5627, CRF22_01A1) includes the 3' end of RT, *int*, *vif*, and the beginning of *vpr*.

Fragment 6 (nt 5628-6052, CRF02_AG) includes the 3' portion of *vpr*, *tat*, and *rev*.

Fragment 7 (nt 6053-6472, CRF22_01A1) includes *vpu* and the 5' portion of *env* gp120.

Fragment 8 (nt 6473-9012, CRF02_AG) includes the 3' portion of *env* gp120, gp41, the second exons of *tat* and *rev*, and the 5' one-third of *nef*.

<u>Supplemental Figure S12: 07CM-663-13</u>
Fragment 1 (nt 827-1448, CRF02_AG) includes *gag* p17 and the 5' one-third of p24.

Fragment 2 (nt 1449-1893, CRF22_01A1) includes the 3' two-thirds of p24, and the 5' half of p2.

Fragment 3 (nt 1894-3715, CRF02_AG) includes the 3' half of *gag* p2, p7, p6, the *pol* protease and the 5' two-thirds of RT.

Fragment 4 (nt 3716-4996, CRF22_01A1) includes the 3' one-third of RT, and most of *int*.

Fragment 5 (nt 4997-5687, CRF02_AG) includes the 3' end of *int*, *vif*, and the 5' portion of *vpr*.

Fragment 6 (nt 5688-8295, CRF22_01A1) includes the 3' portion of *vpr, tat, rev, vpu*, *env* gp120, and the 5' half of *env* gp41.

Fragment 7 (nt 8296-9012, CRF02_AG) includes the 3' half of gp41, the second exons of *tat* and *rev*, and the 5' one-third of *nef*.

Supplemental Figure S13: 08CM-867-10

Fragment 1 (nt 827-4836, subtype H) includes *gag*, the *pol* protease, RT, and the 5’ portion of *int*.

Fragment 2 (nt 4837-6116, subtype A) includes the 3’ portion of *int*, *vif, vpr, tat, rev* and the 5’ portion of *vpu*.

Fragment 3 (nt 6117-7670, subtype H) includes the 3’ portion of *vpu* and most of *env* gp120.

Fragment 4 (nt 7671-8295, subtype A) includes the 3’ end of *env* gp120 and the 5’ portion of *env* gp41.

Fragment 5 (nt 8296-9012, subtype H) includes the 3’ portion of gp41, the second exons of *tat* and *rev*, and the 5’ one-third of *nef*.

Supplemental Figure S14: 08CM-1252-11

Fragment 1 (nt 827-2583, CRF11_cpx) includes *gag*, the *pol* protease, and the beginning of RT.

Fragment 2 (nt 2584-2863, subtype F2) includes the 5’ one-sixth of RT.

Fragment 3 (nt 2864-4871, CRF11_cpx) includes the 3’ portion of RT and most of *int*.

Fragment 4 (nt 4872-5401, subtype F2) includes the 3’ end of *int* and the 5’ two-thirds of *vif*.

Fragment 5 (nt 5402-9012, CRF11_cpx) includes the 3’ portion of *vif*, *vpr*, *tat, rev*, *vpu*, *env*, the second exons of *tat* and *rev*, and the 5’ one-third of *nef*.

Supplemental Figure S15: 11CM-CHU3903

Fragment 1 (nt 827-1981, CRF22_01A1) includes *gag* p17, p24, p2, and the 5’ half of p7.

Fragment 2 (nt 1982-2958, subtype F2) includes the 3’ half of *gag* p7, p6, the *pol* protease, and the 5’ portion of RT.

Fragment 3 (nt 2959-9012, CRF22_01A1) includes the 3’ portion of RT, *int, vif, vpr, tat, rev, vpu, env,* the second exons of *tat* and *rev*, and the 5’ one-third of *nef*.

Supplemental Figure S16: 11CM-CHU2727

Fragment 1 (nt 827-6090, subtype A) includes *gag, pol, vif, vpr, tat, rev*, and the 5’ portion of *vpu*.

Fragment 2 (nt 6091-8335, subtype H) includes the 3’ portion of *vpu*, *env* gp120, and the 5’ half of *env* gp41.

Fragment 3 (nt 8336-9012, subtype A) includes the 3’ half of gp41, the second exons of *tat* and *rev*, and the 5’ one-third of *nef*
