# Supplementary figures and images for: Utility of Metagenomic Next-Generation Sequencing for Characterization of HIV and Human Pegivirus Diversity
Source: PLoS One. 2015 Nov 23;10(11):e0141723. doi: 10.1371/journal.pone.0141723 (PMC4658132; doi:10.1371/journal.pone.0141723)

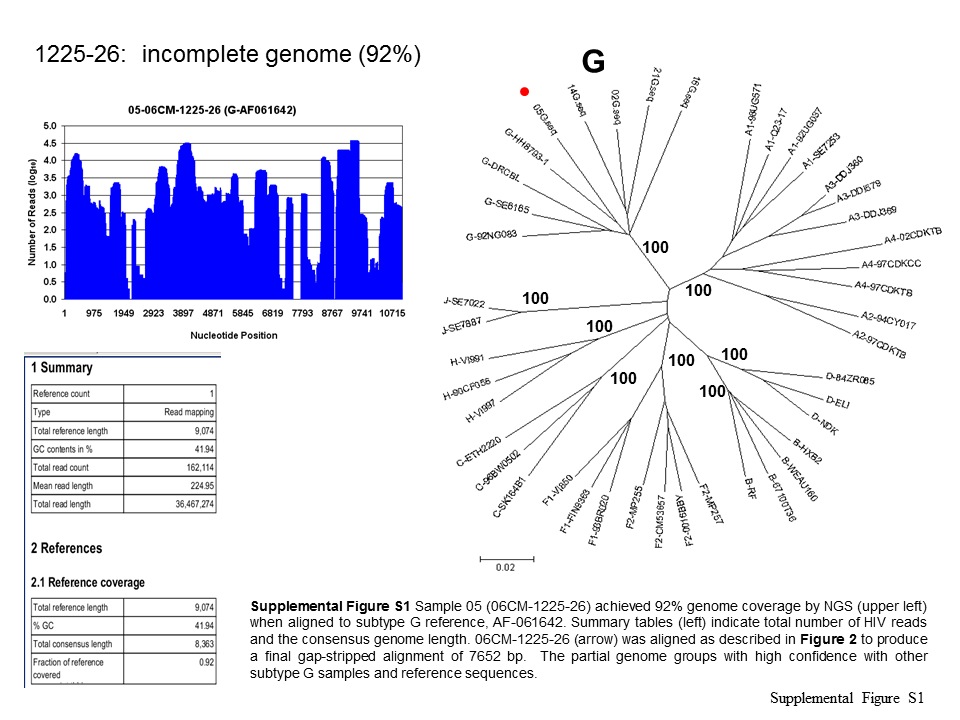

Supplement: S1 Fig — (TIF) [file pone.0141723.s002.TIF]

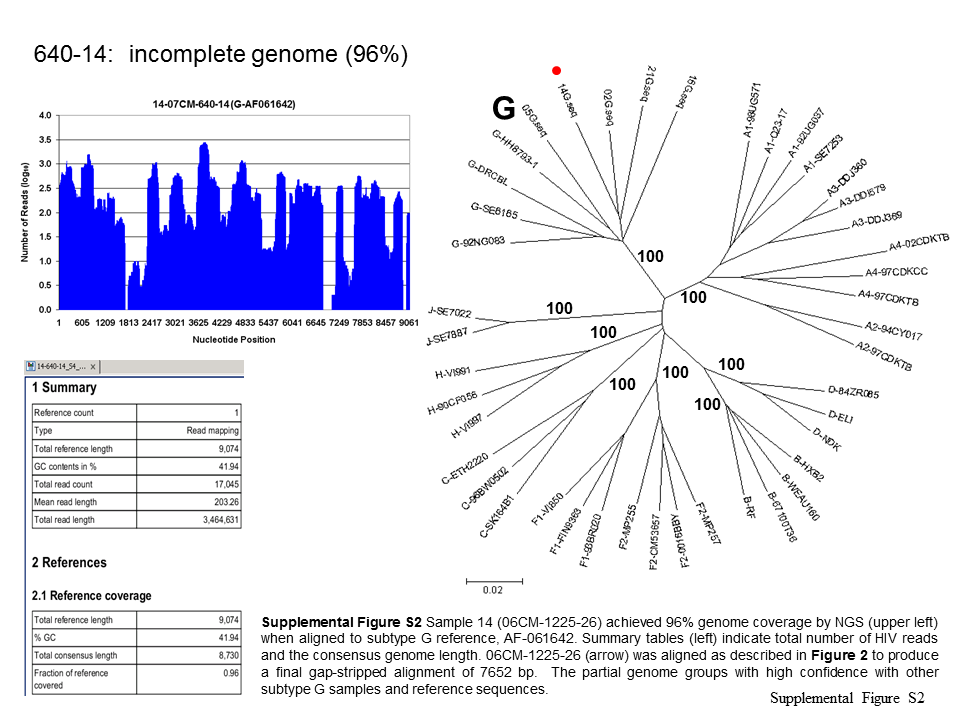

Supplement: S2 Fig — (TIF) [file pone.0141723.s003.TIF]

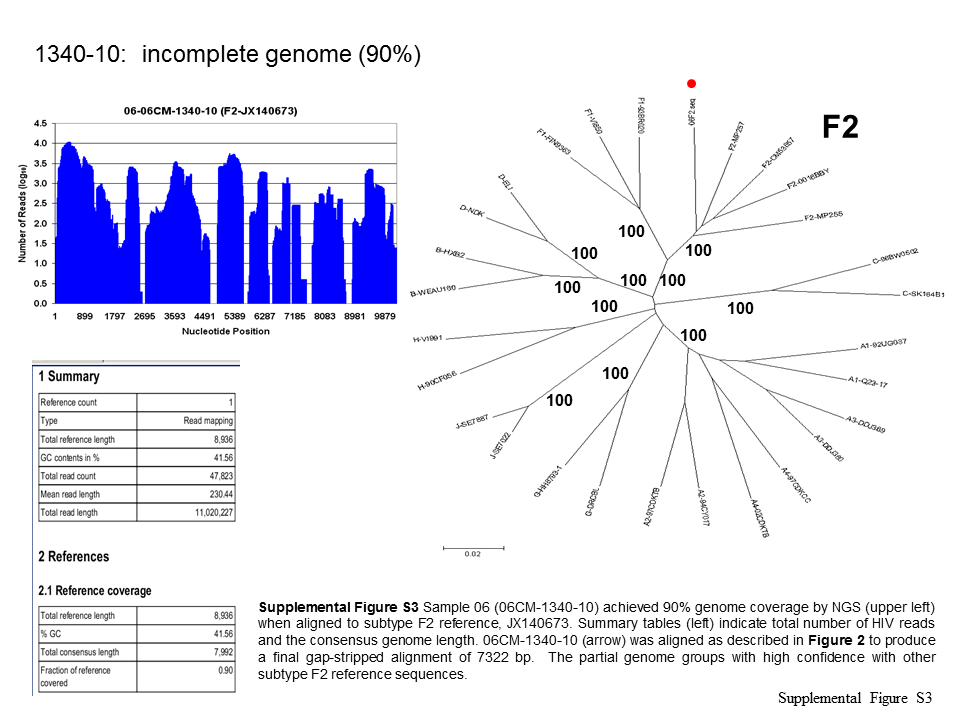

Supplement: S3 Fig — (TIF) [file pone.0141723.s004.TIF]

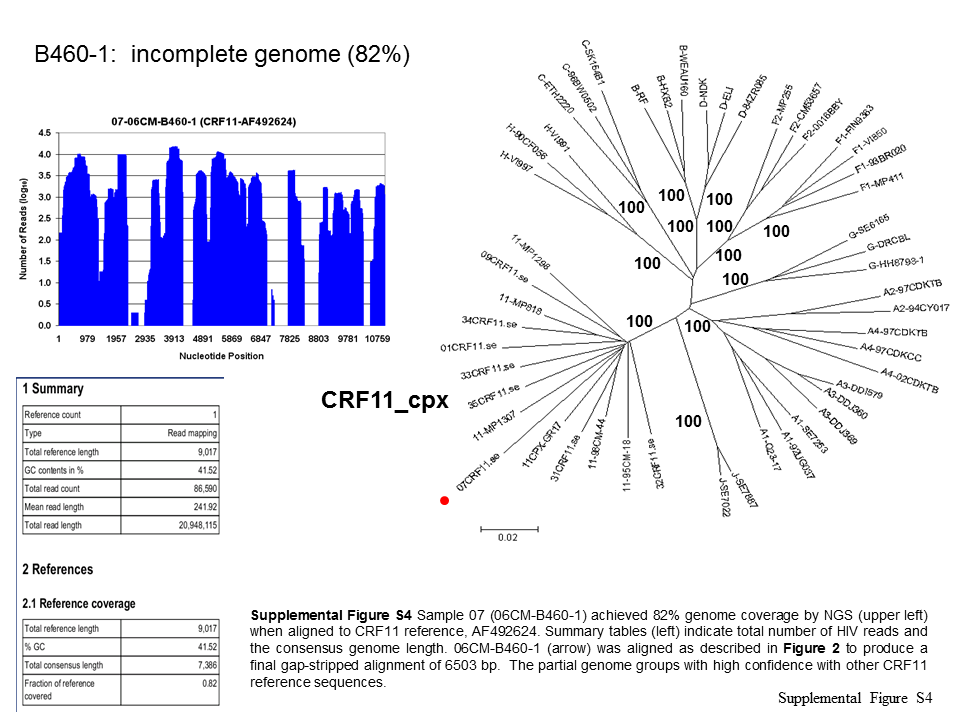

Supplement: S4 Fig — (TIF) [file pone.0141723.s005.TIF]

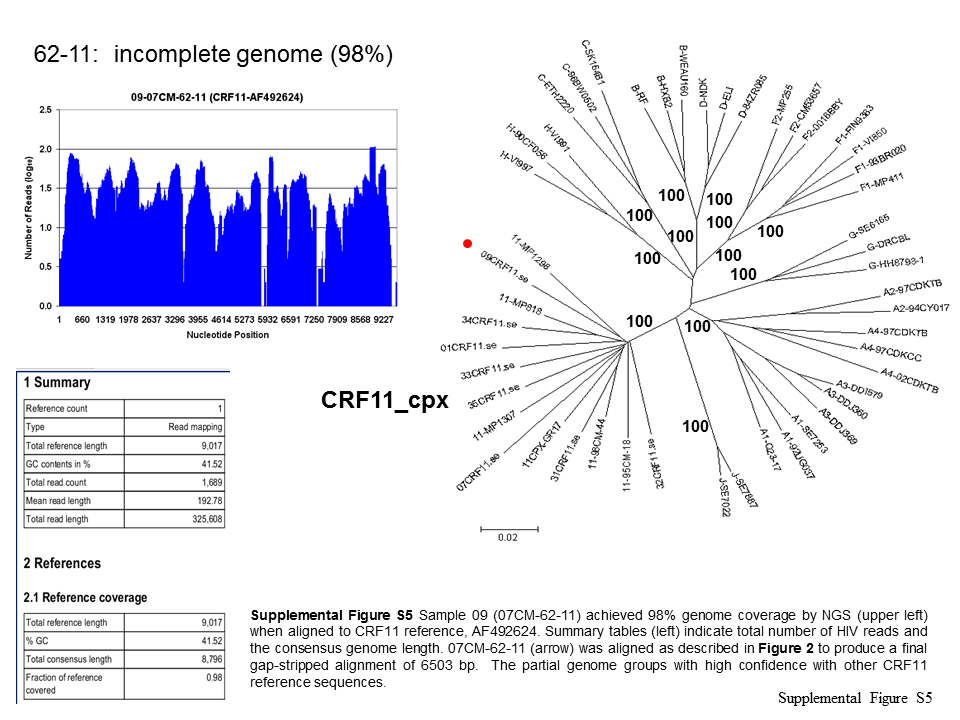

Supplement: S5 Fig — (TIF) [file pone.0141723.s006.TIF]

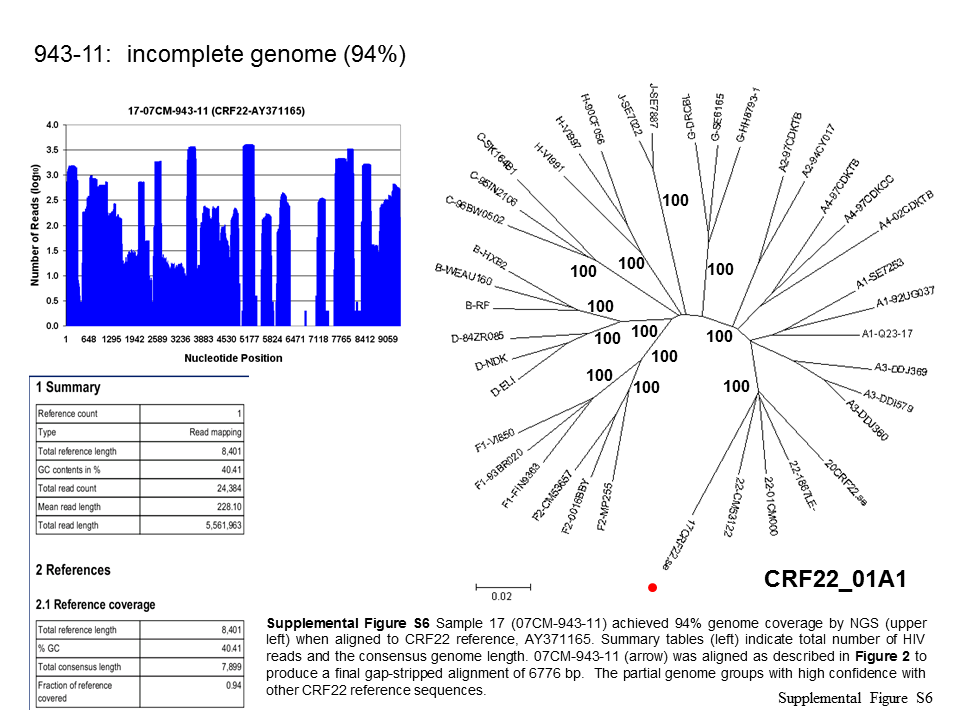

Supplement: S6 Fig — (TIF) [file pone.0141723.s007.TIF]

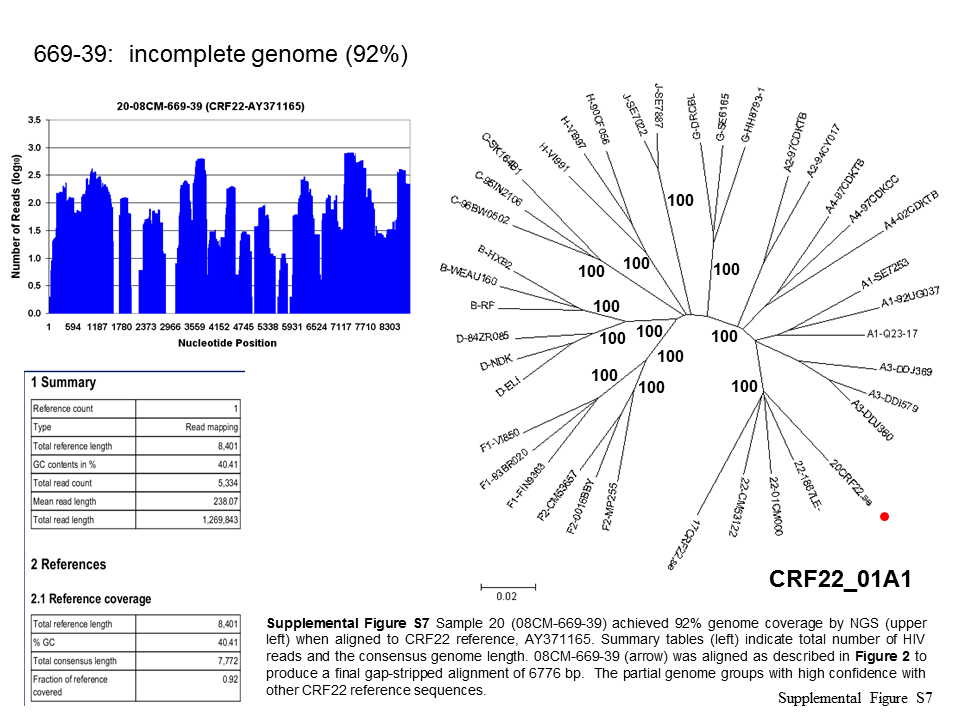

Supplement: S7 Fig — (TIF) [file pone.0141723.s008.TIF]

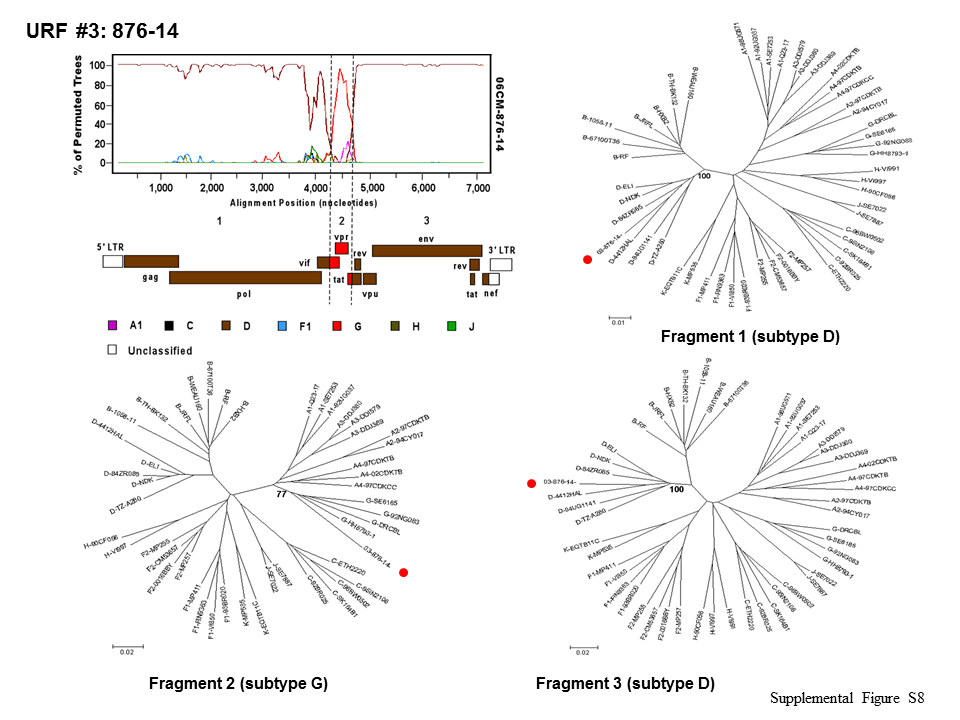

Supplement: S8 Fig — (TIF) [file pone.0141723.s009.TIF]

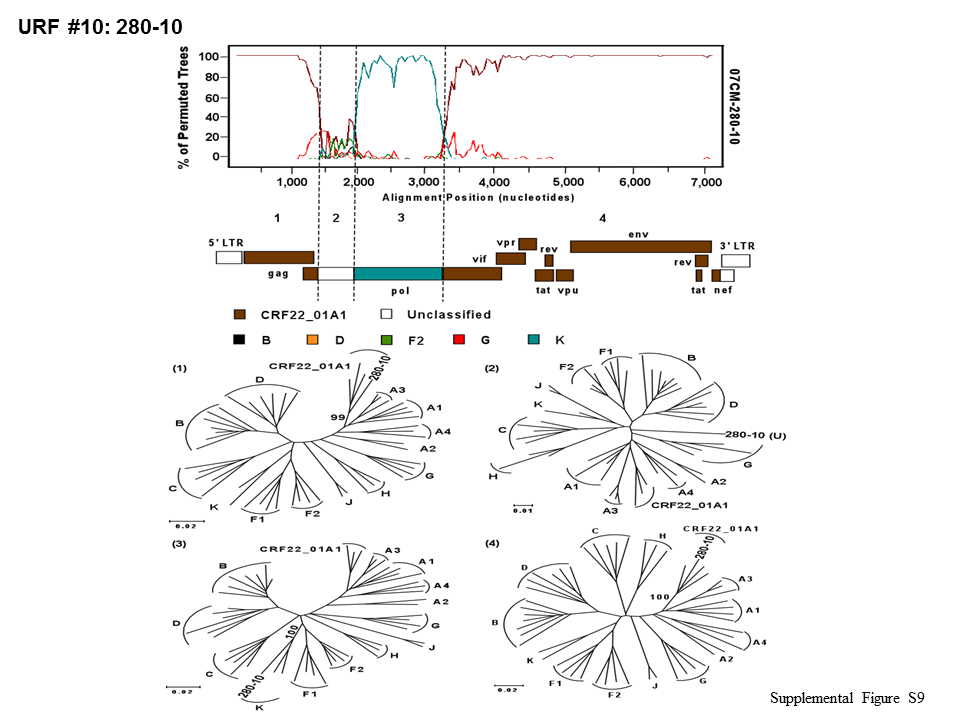

Supplement: S9 Fig — (TIF) [file pone.0141723.s010.TIF]

**URF #12: 469-66**

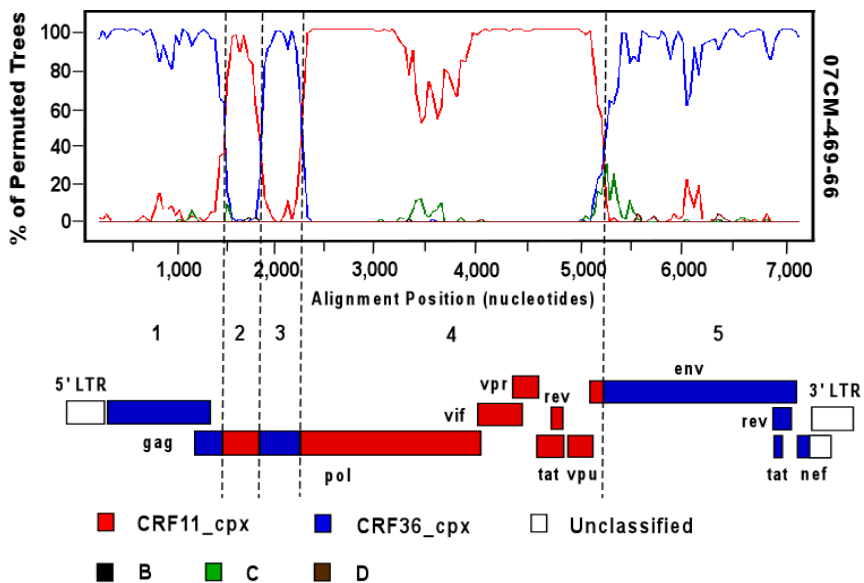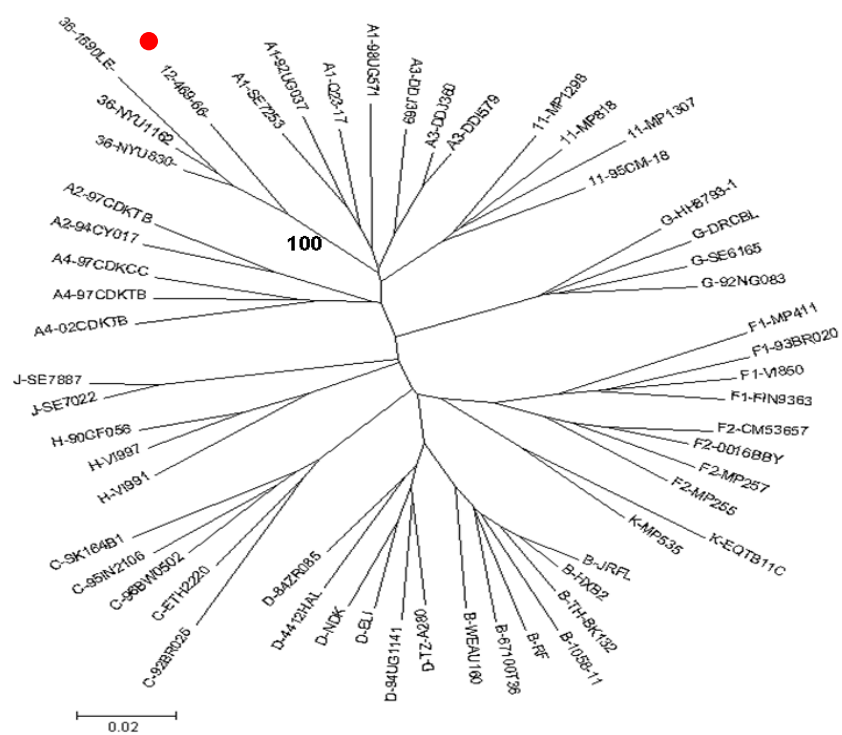

### Fragment 1 (CRF36\_cpx)

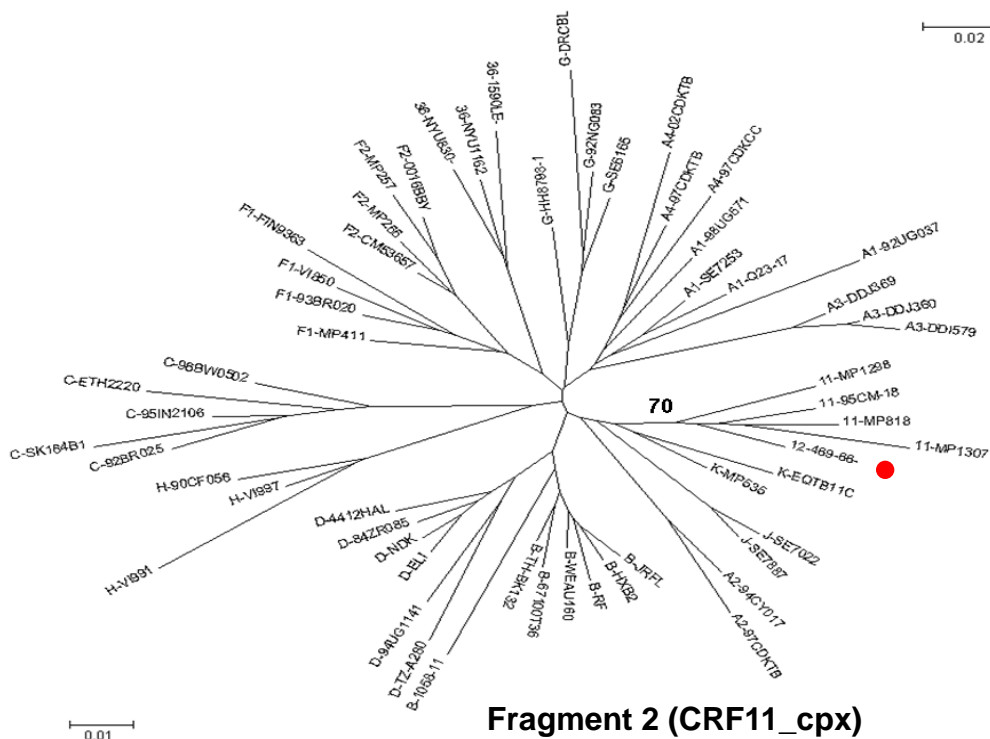

## Fragment 2 (CRF11\_cpx)

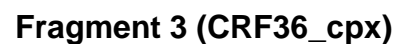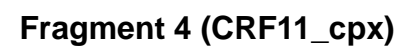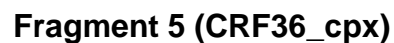

Supplement: S10 Fig — (PDF) [file pone.0141723.s011.pdf]

# URF #13: 567-16

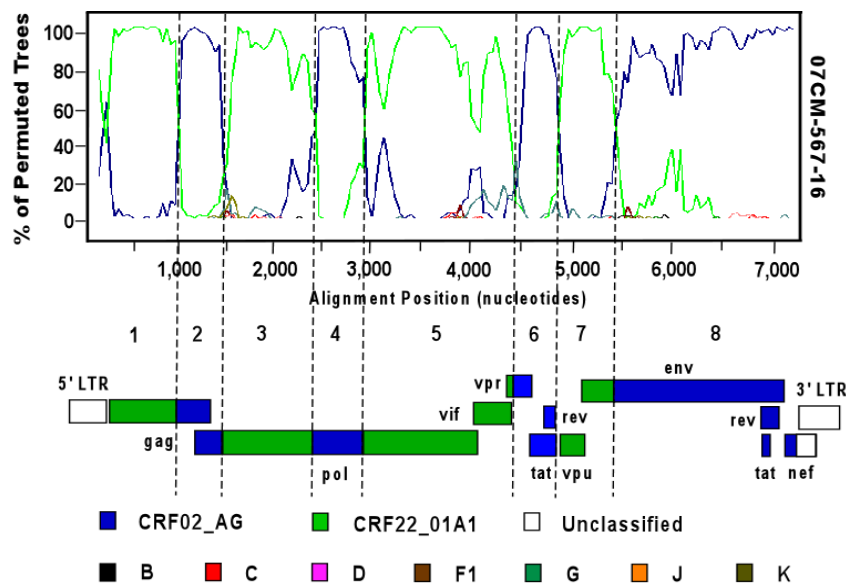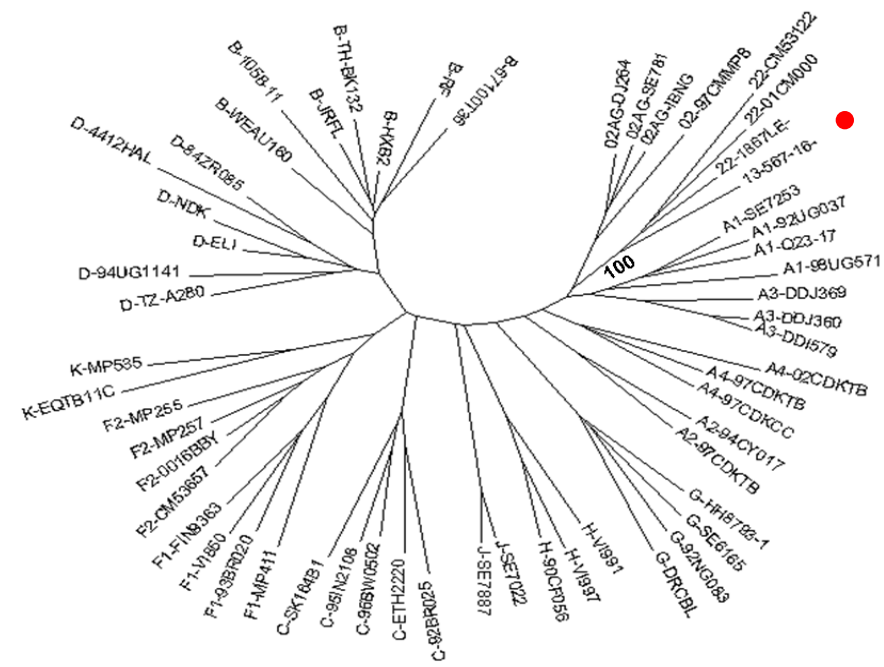

Fragment 1 (CRF22\_01A1)

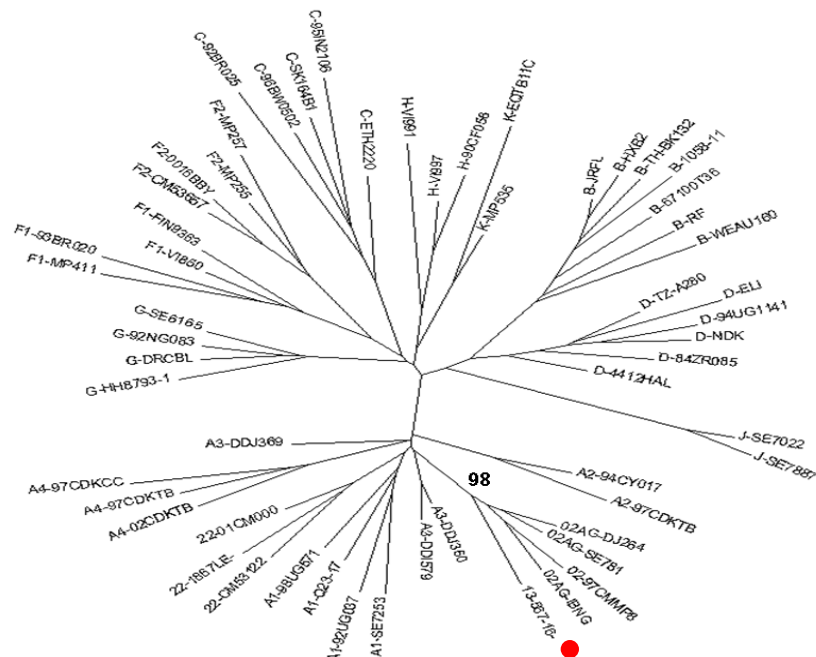

Fragment 2 (CRF02\_AG)

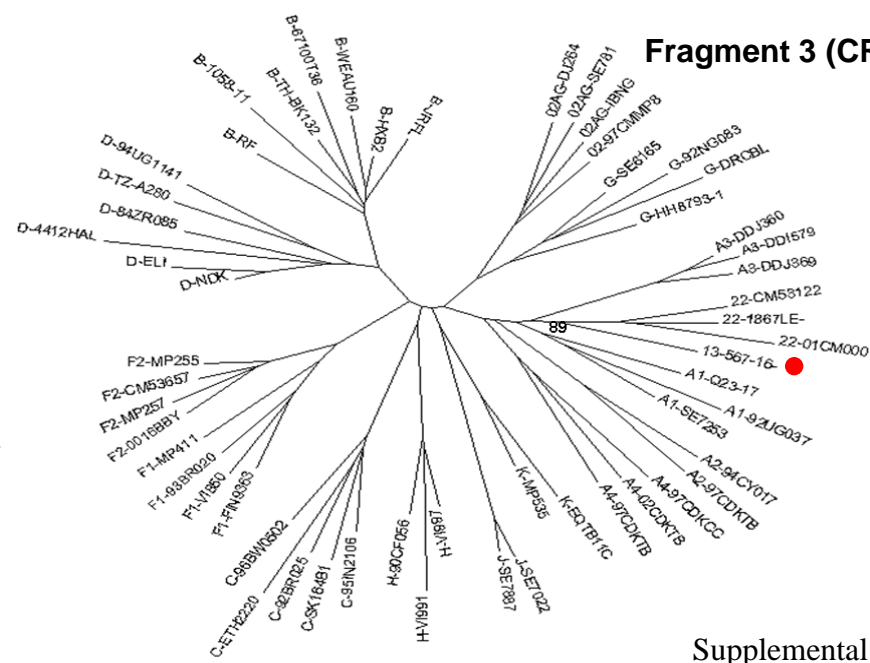

Fragment 3 (CRF22\_01A1)

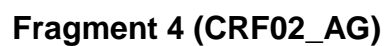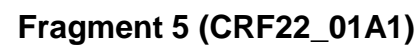

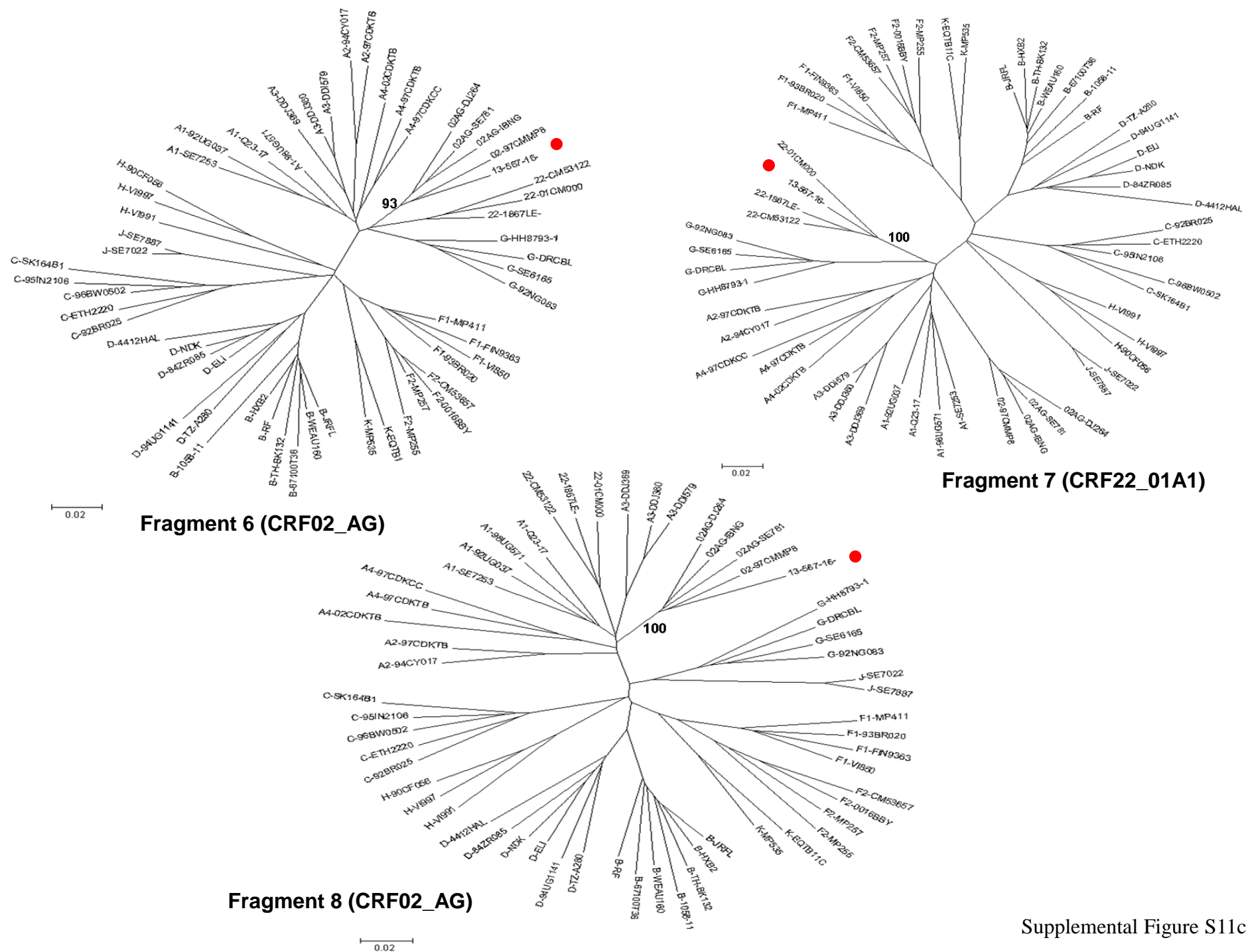

Supplement: S11 Fig — (PDF) [file pone.0141723.s012.pdf]

# URF #15: 663-13

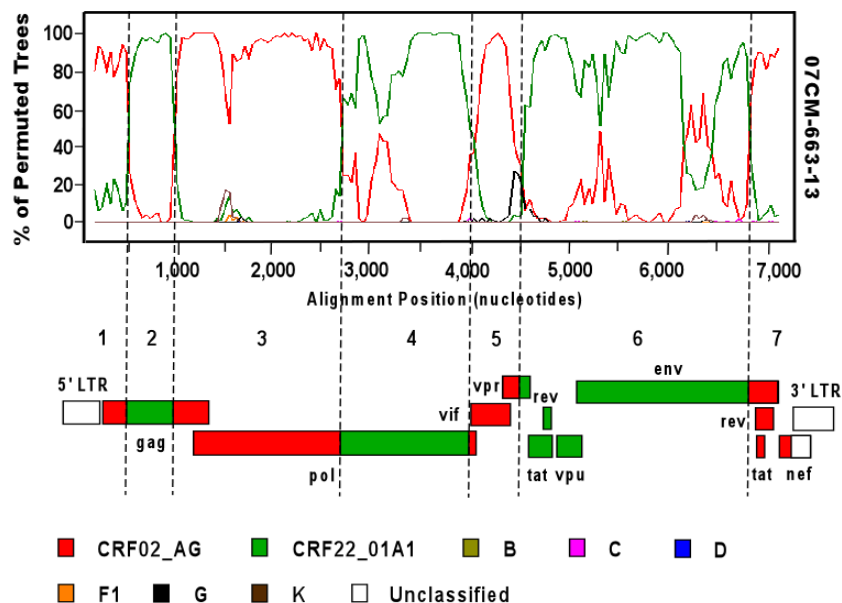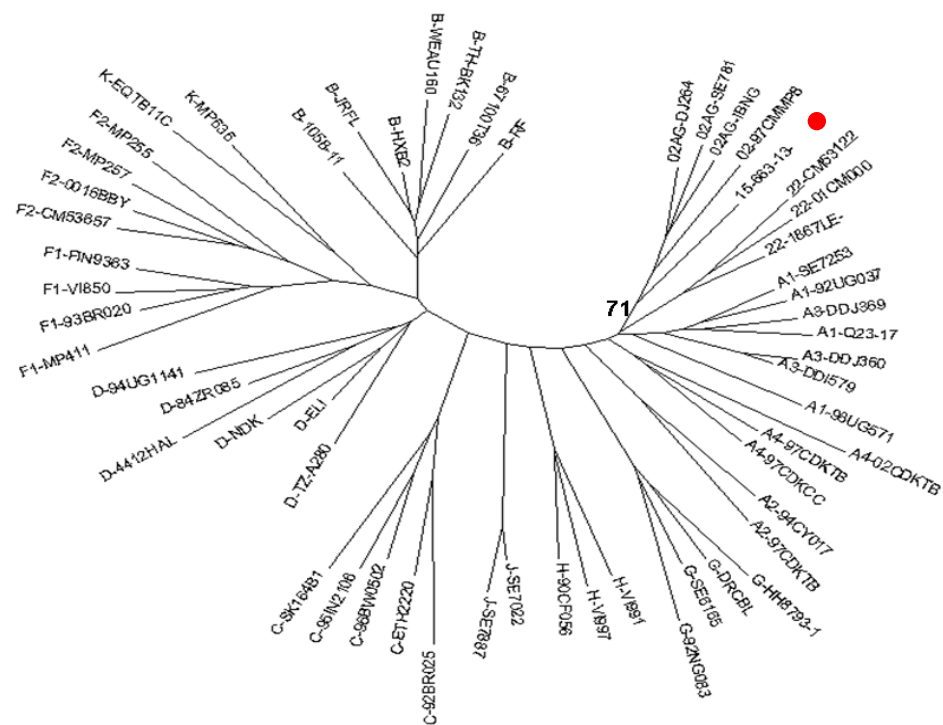

Fragment 1 (CRF02\_AG)

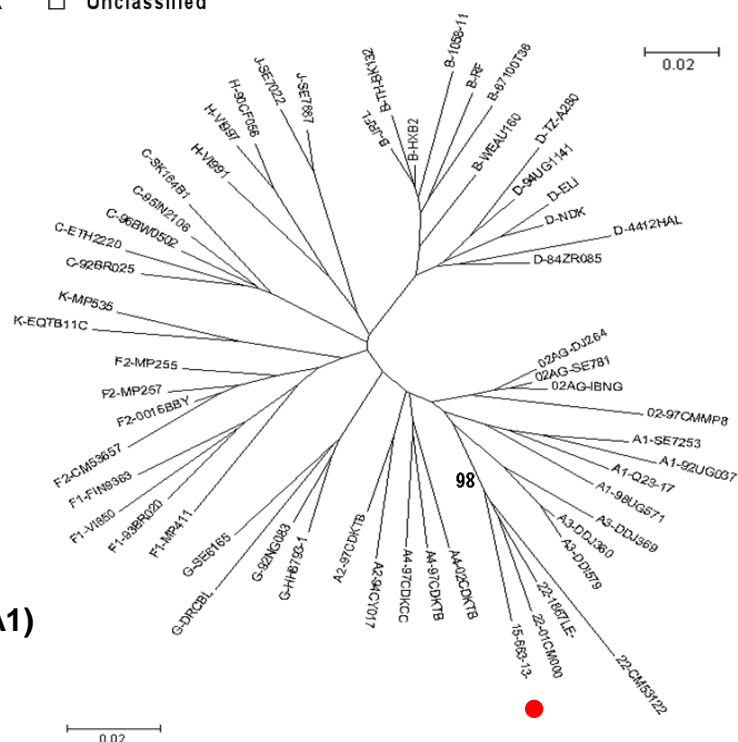

Fragment 2 (CRF22\_01A1)

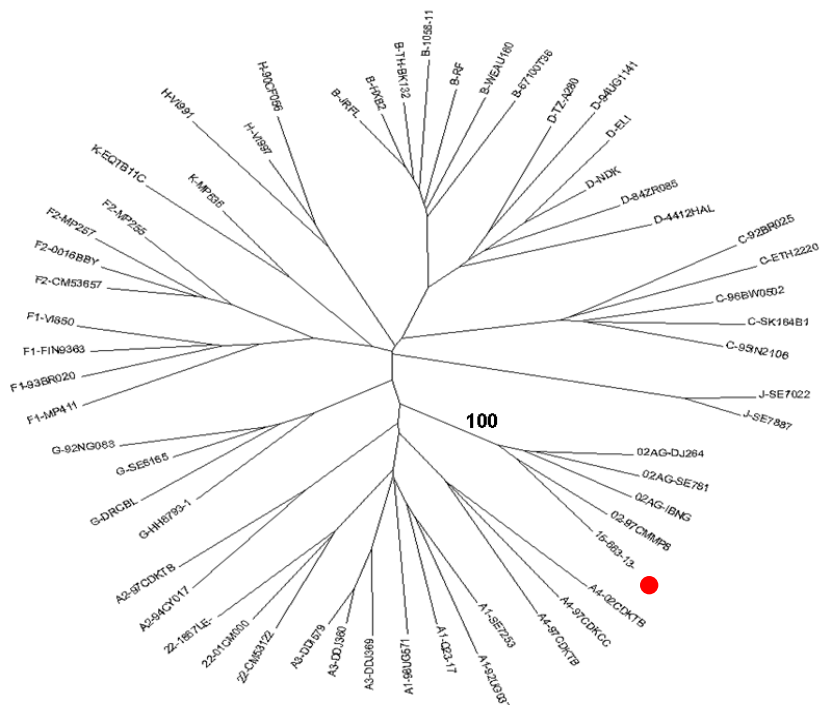

Fragment 3 (CRF02\_AG)

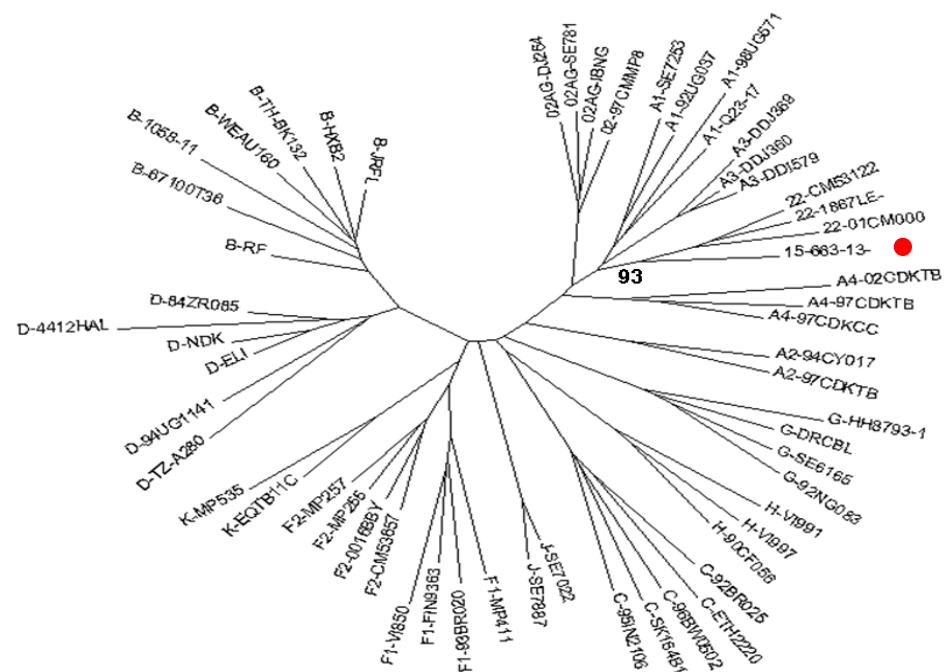

Fragment 4 (CRF22\_01A1)

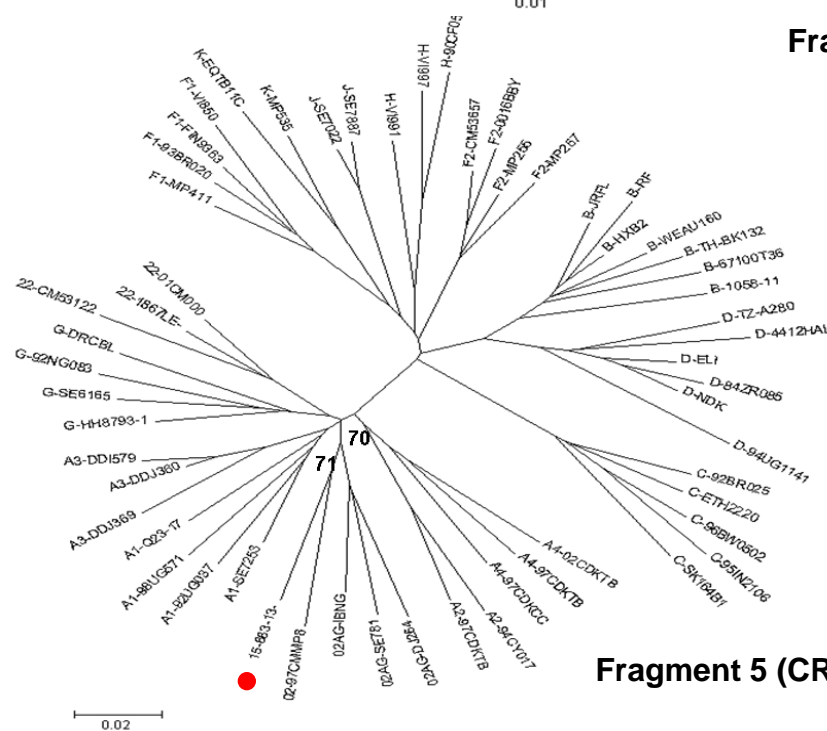

Fragment 5 (CRF02\_AG)

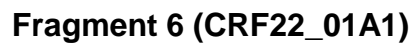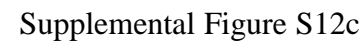

Supplement: S12 Fig — (PDF) [file pone.0141723.s013.pdf]

# URF #23: 867-10

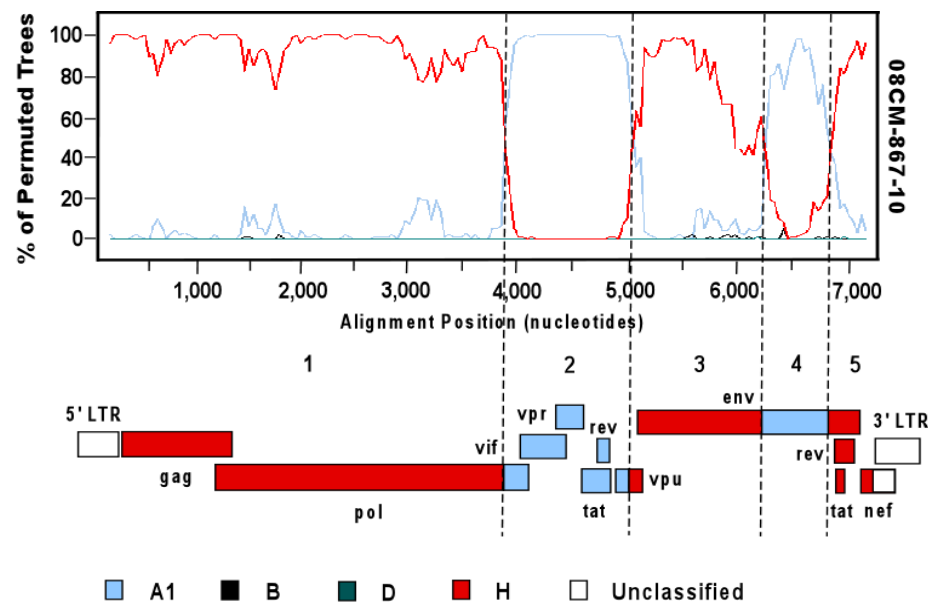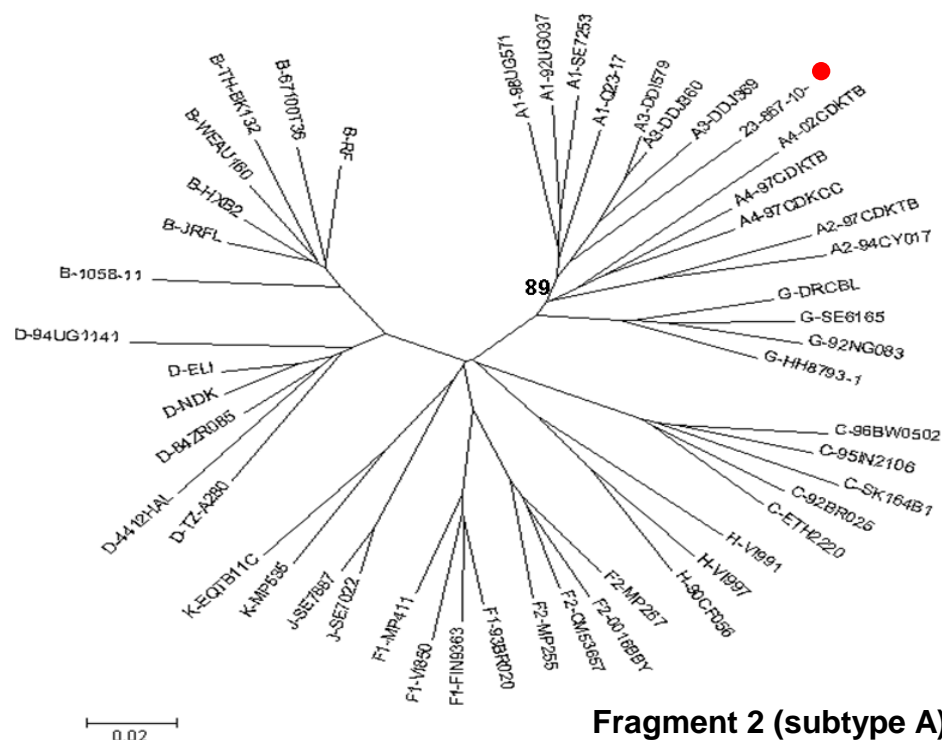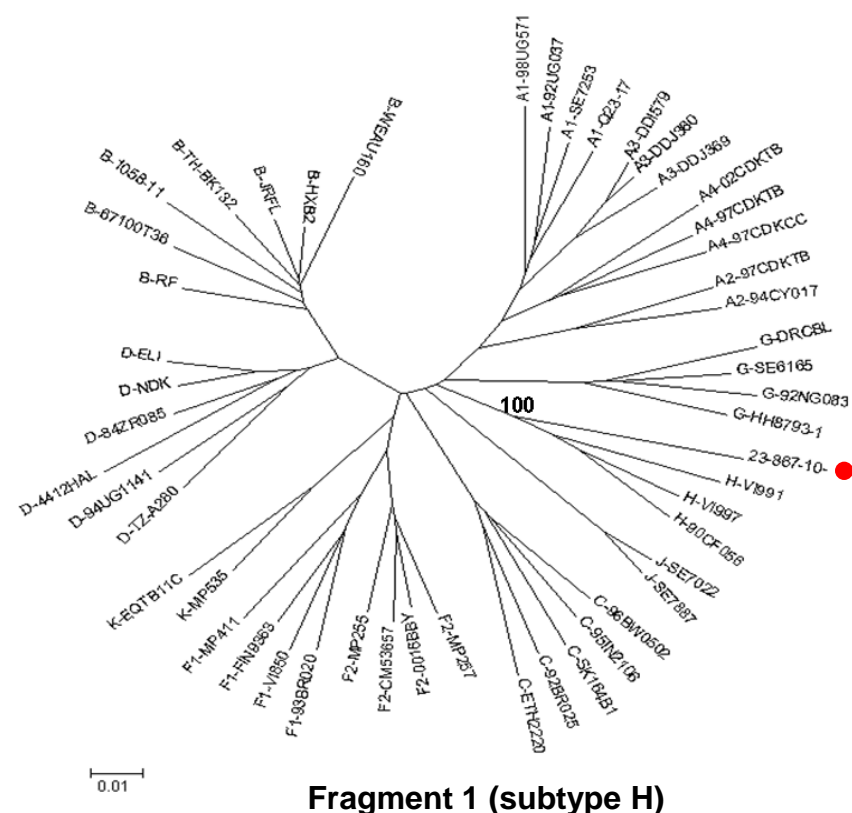

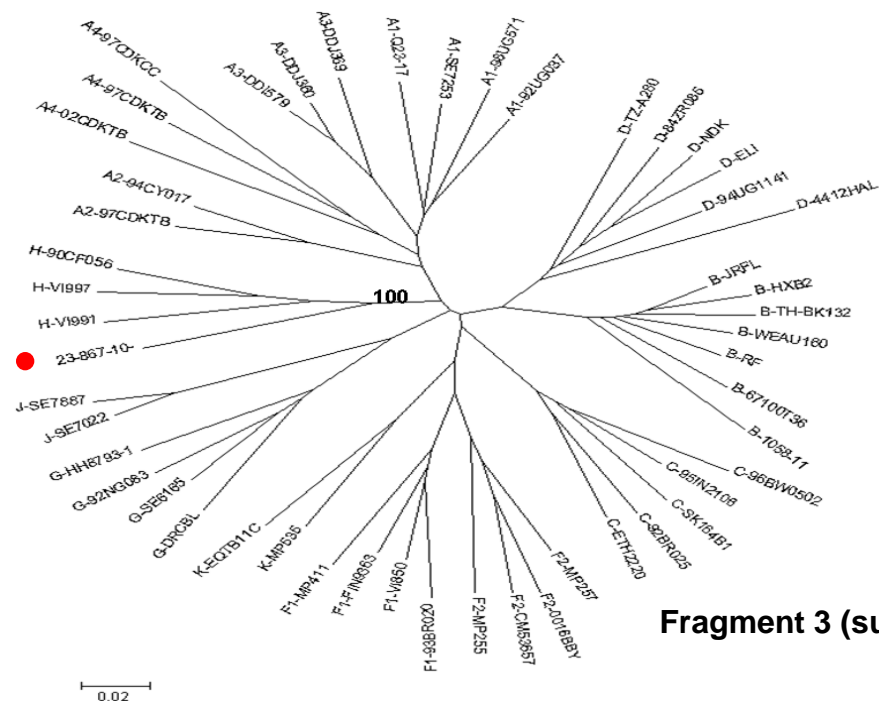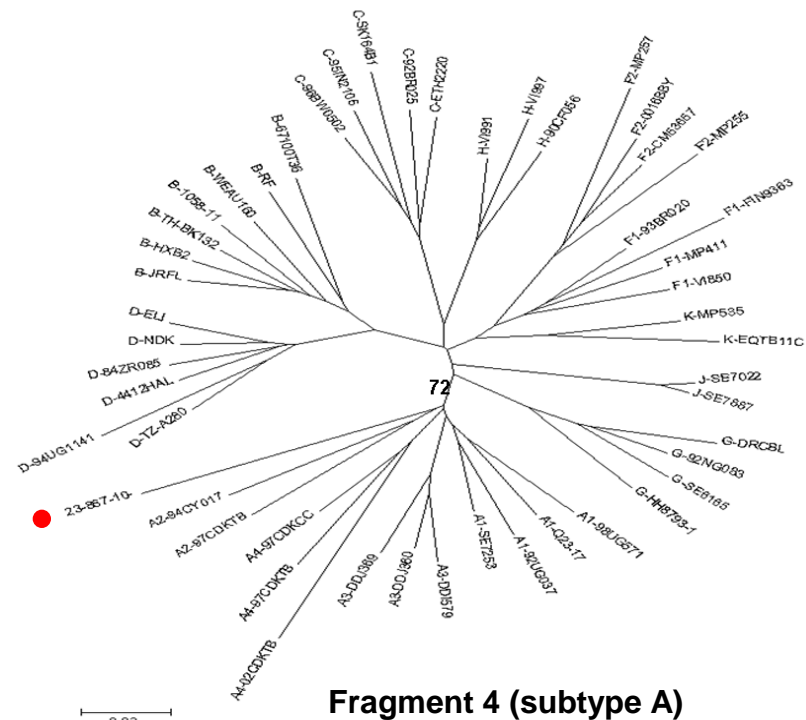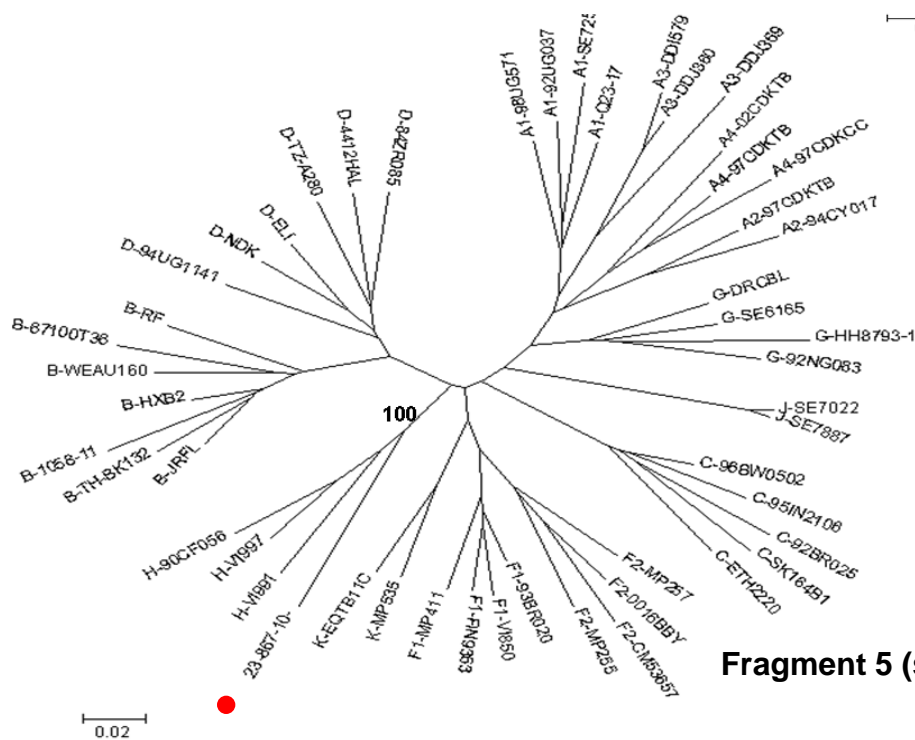

Supplement: S13 Fig — (PDF) [file pone.0141723.s014.pdf]

# URF #25: 1252-11

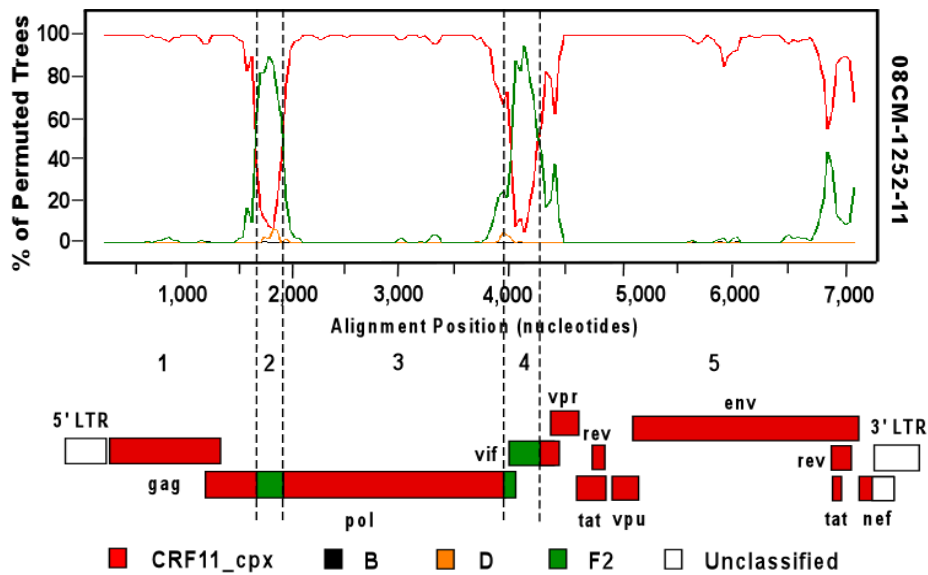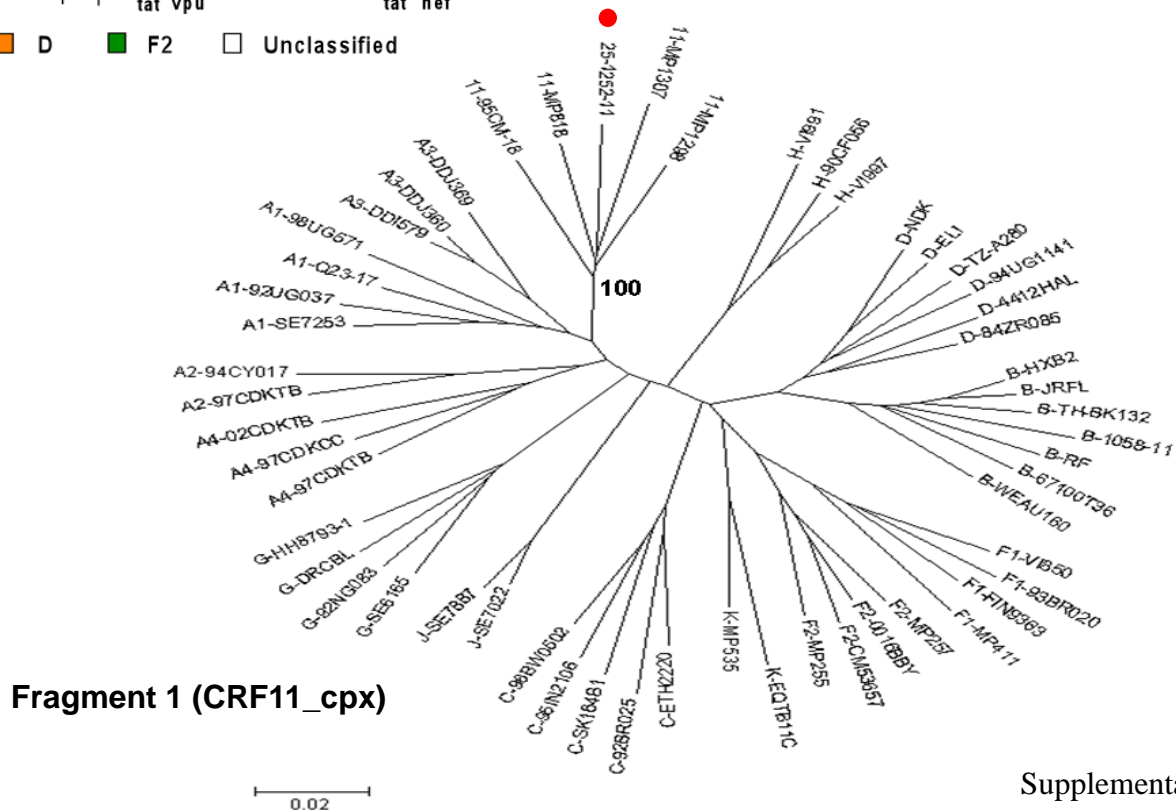

## Fragment 2 (subtype F2)

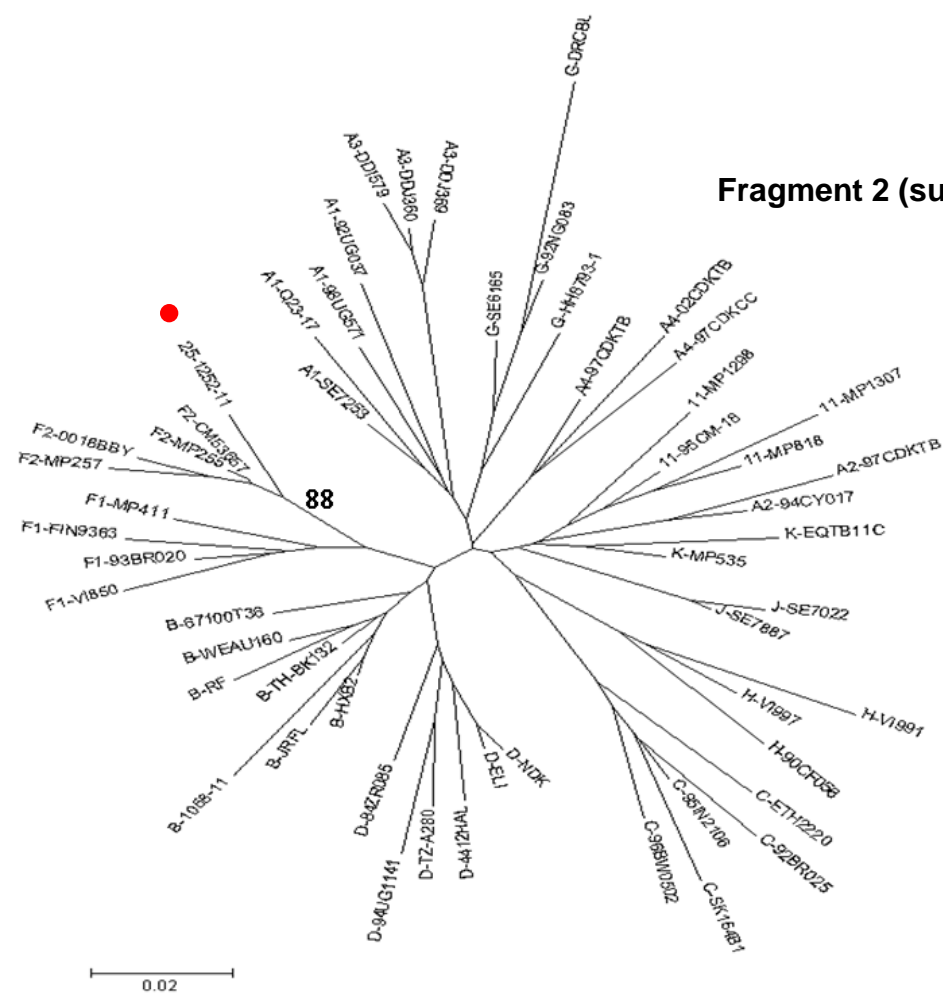

## Fragment 3 (CRF11\_cpx)

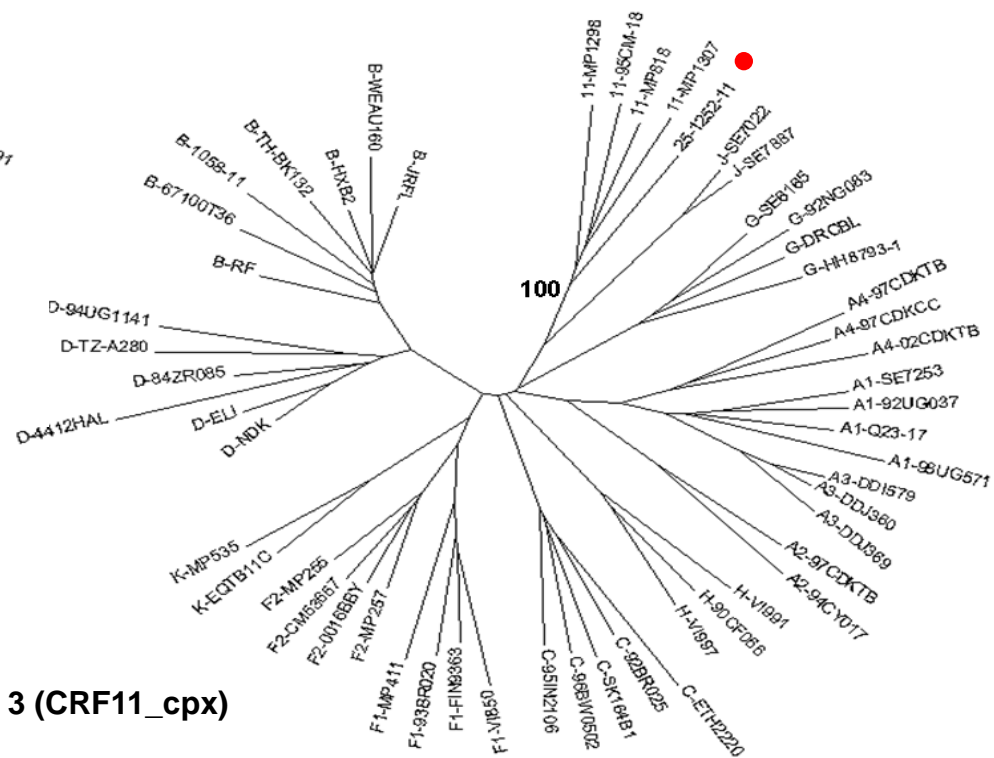

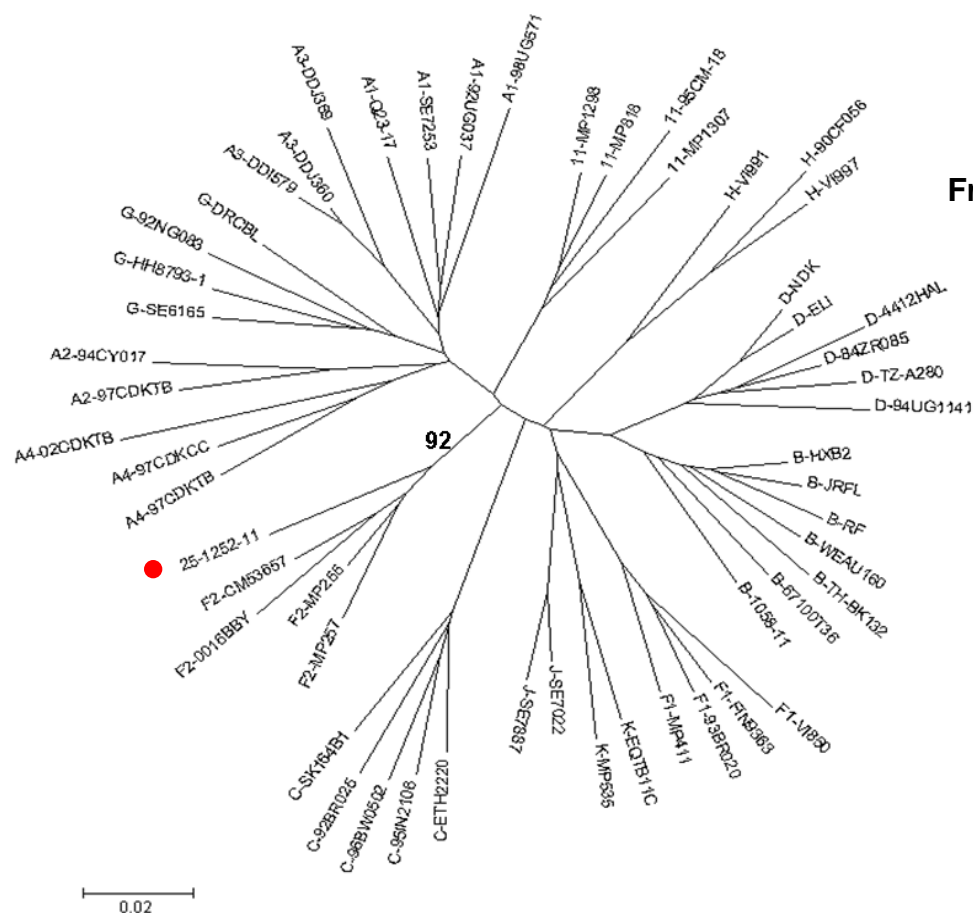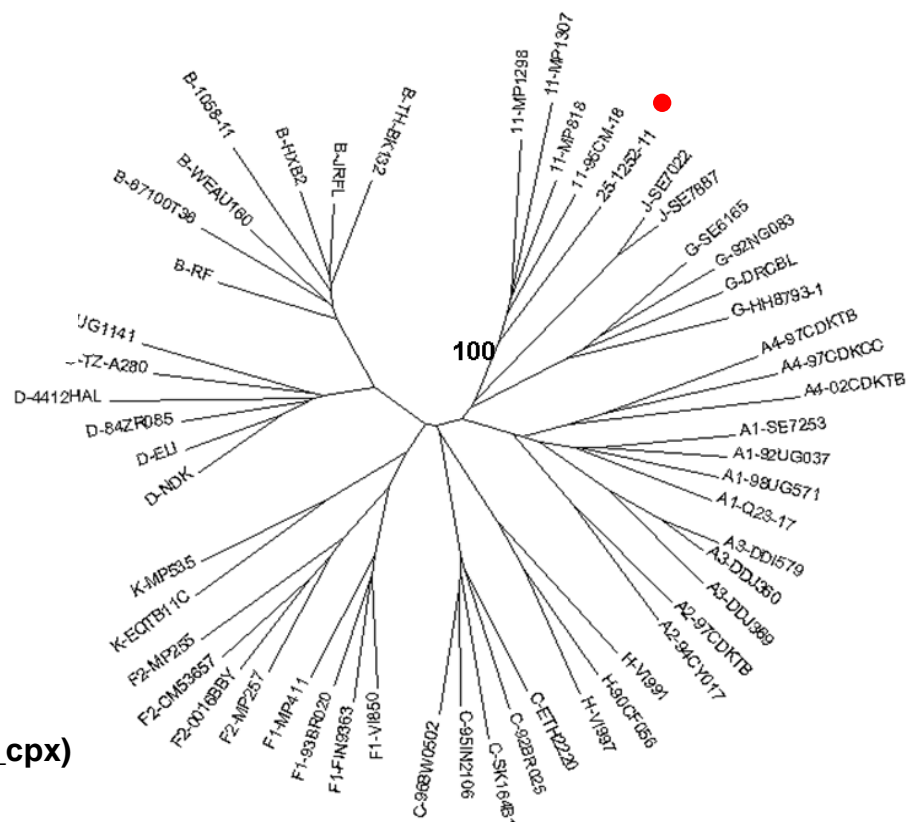

Supplement: S14 Fig — (PDF) [file pone.0141723.s015.pdf]

# URF #28: CHU3903

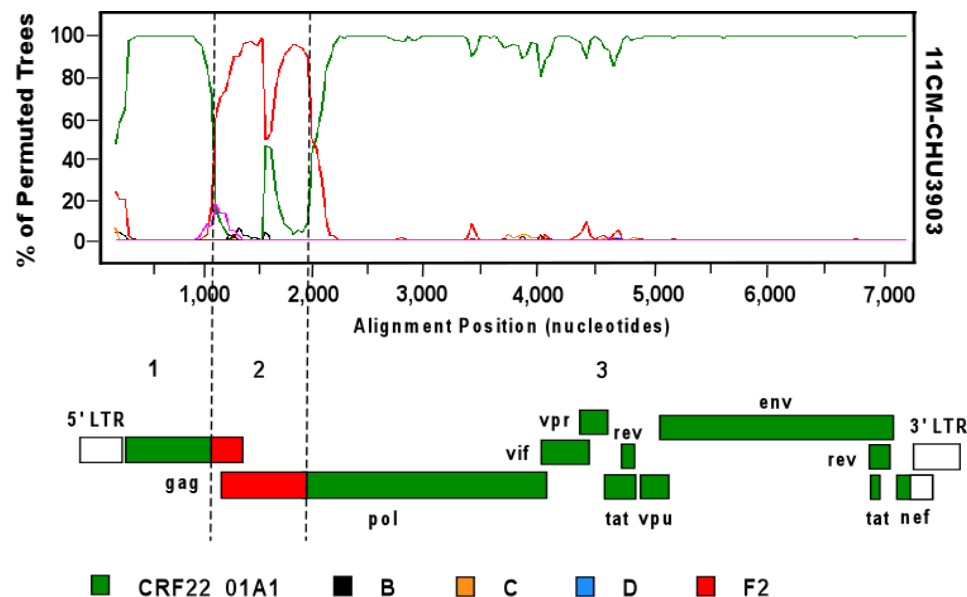

Fragment 1 (CRF22\_01A1)

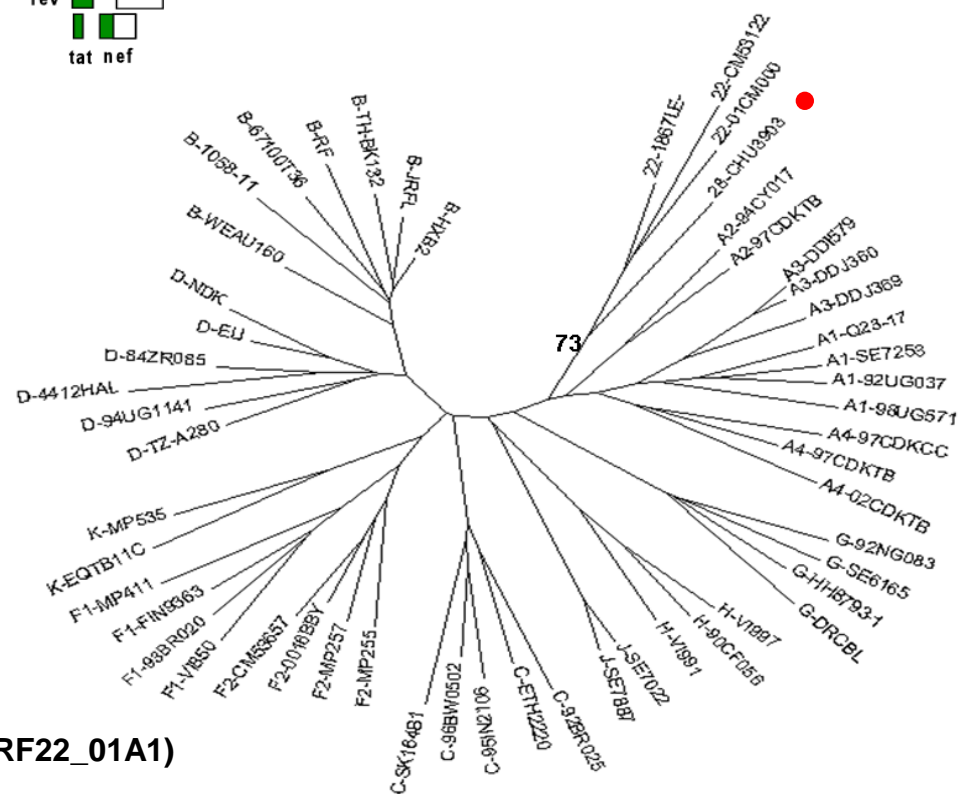

## Fragment 2 (subtype F2)

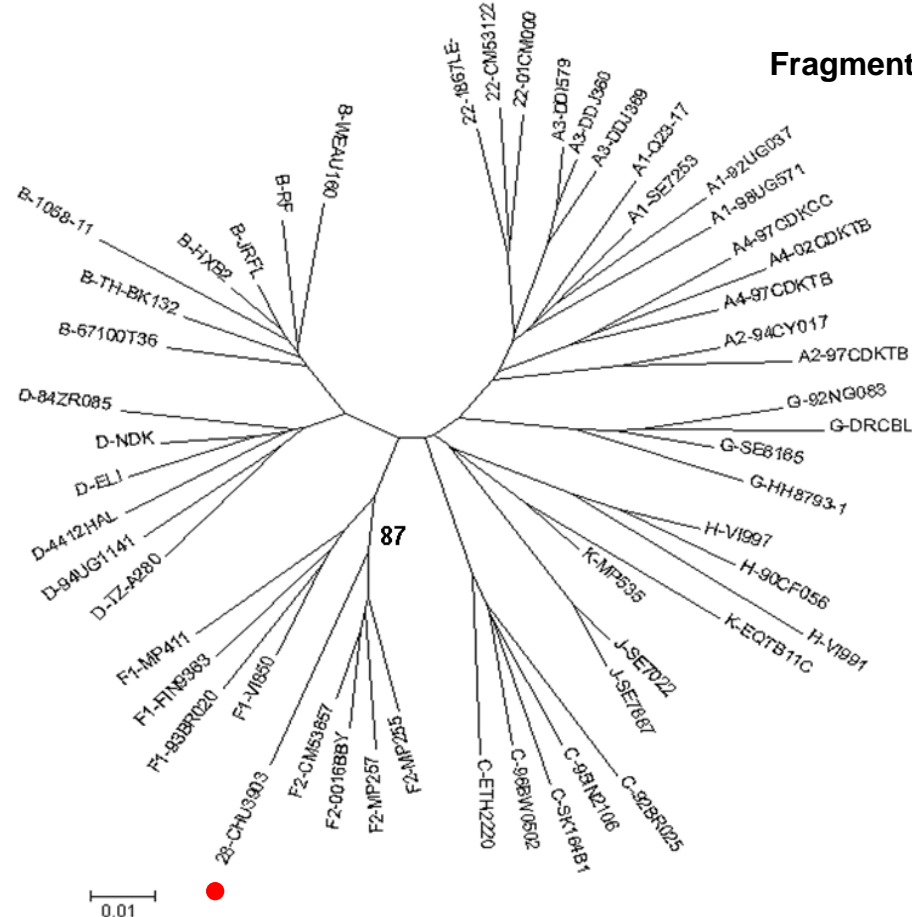

## Fragment 3 (CRF22\_01A1)

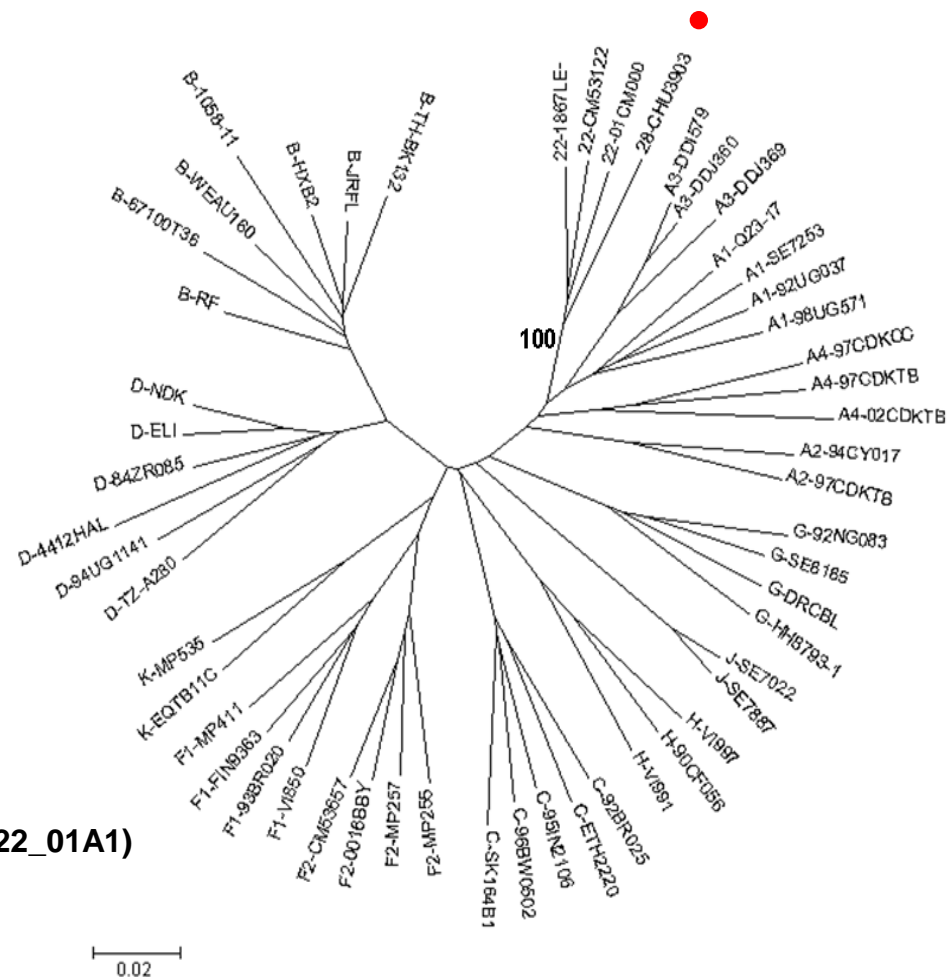

Supplement: S15 Fig — (PDF) [file pone.0141723.s016.pdf]

URF #29: CHU2727

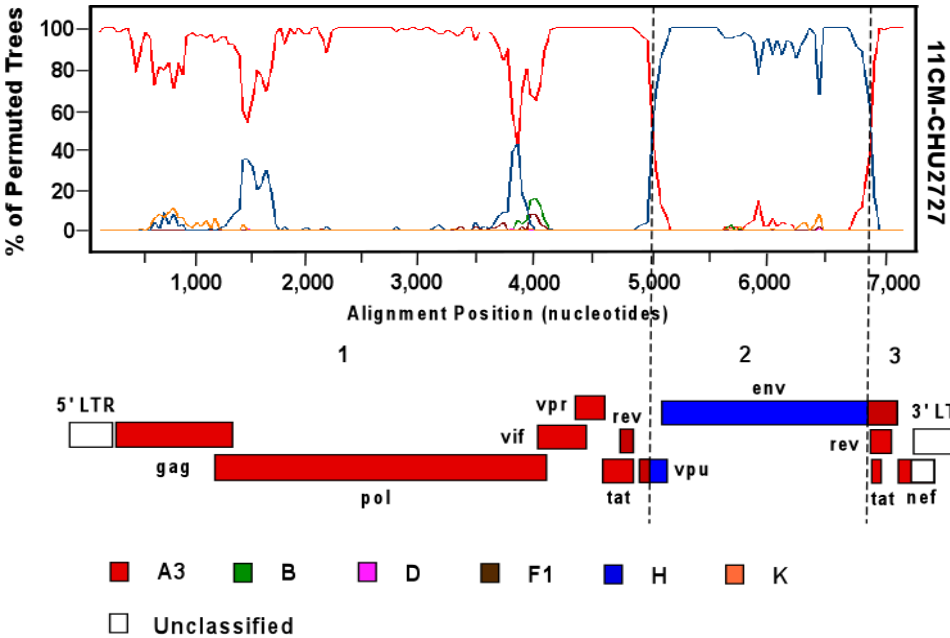

Fragment 1 (subtype A)

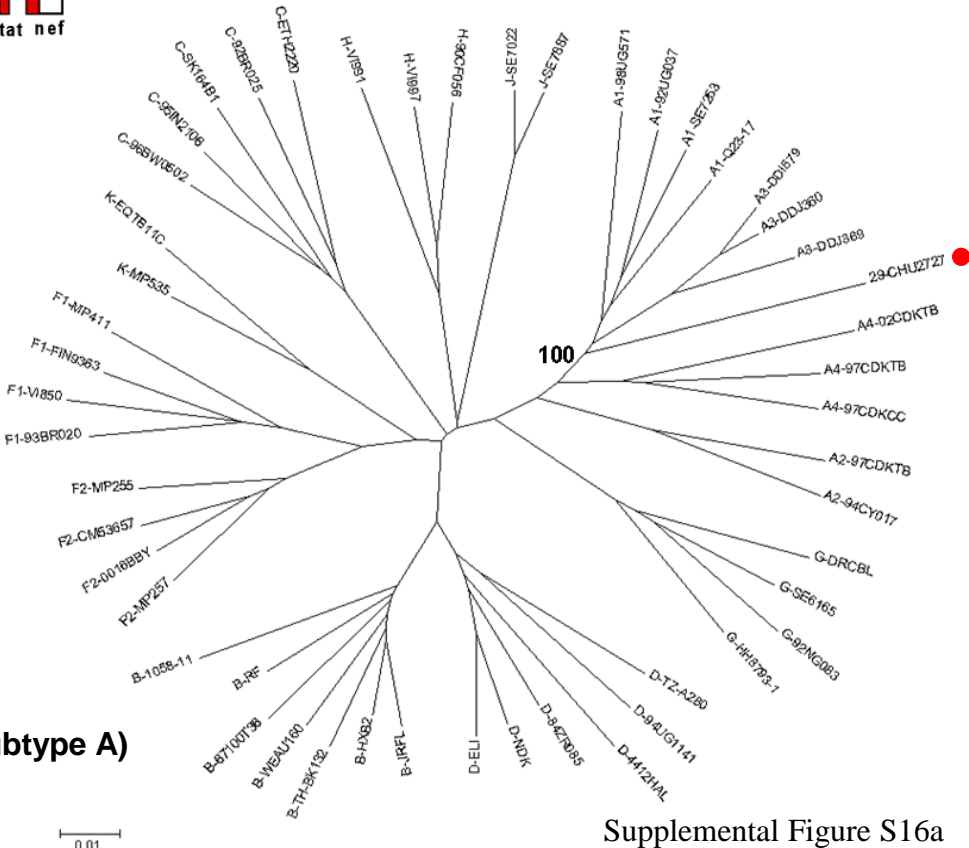

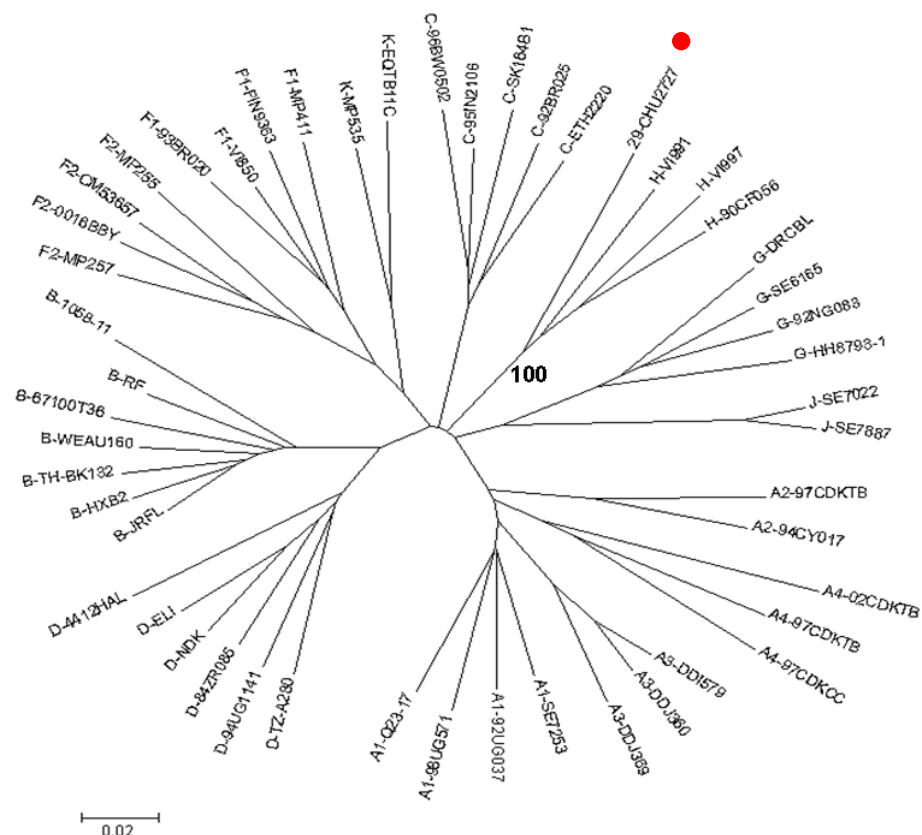

**Fragment 2 (subtype H)**

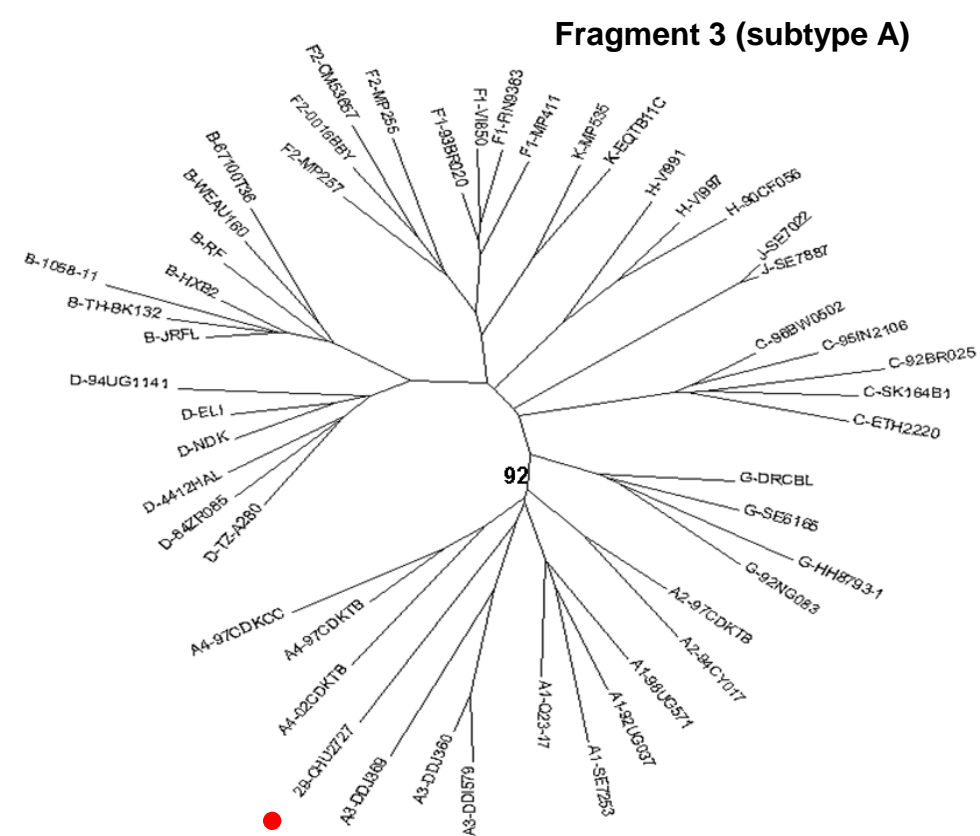

**Fragment 3 (subtype A)**

Supplement: S16 Fig — (PDF) [file pone.0141723.s017.pdf]
