# Supplementary material for: Utility of Metagenomic Next-Generation Sequencing for Characterization of HIV and Human Pegivirus Diversity
Source: PLoS One. 2015 Nov 23;10(11):e0141723. doi: 10.1371/journal.pone.0141723 (PMC4658132; doi:10.1371/journal.pone.0141723)
Supplement: S1 Table — (PDF) [file pone.0141723.s018.pdf]

Specimen ID	Donor	
	sex	age
263-26	na	na
740-10	na	na
876-33	na	na
1130-39	M	38
1225-26	na	na
1340-10	na	na
B460-1	na	na
46-10	F	32
62-11	F	22
280-10	M	46
419-33	F	39
469-66	M	36
567-16	M	40
640-14	na	na
663-13	M	30
920-49	M	37
943-11	M	36
38-38	M	29
228-10	M	35
669-39	M	38
789-10	M	39
833-62	M	36
867-10	M	47
886-24	F	23
1252-11	M	19
1156-26	na	na
B4043-15	na	na
CHU3903	na	na
CHU2727	na	na
CHU2801	na	na
A1575	na	na
A1774	na	na
119-28	M	33
260-50	na	na
1230-24	M	37

Supplemental Table S1. Cameroonian blood donor (specimen ID) demographic data (na; not available).
